# Convergent evolution in toxin detection and resistance provides evidence for conserved bacterial–fungal interactions

**DOI:** 10.1073/pnas.2304382121

**Published:** 2024-08-01

**Authors:** Stephen K. Dolan, Ashley T. Duong, Marvin Whiteley

**Affiliations:** ^a^School of Biological Sciences, Georgia Institute of Technology, Atlanta, GA 30310; ^b^Department of Genetics and Biochemistry, Eukaryotic Pathogens Innovation Center, Clemson University, Clemson, SC 29634; ^c^Emory-Children’s Cystic Fibrosis Center, Atlanta, GA 30310; ^d^Center for Microbial Dynamics and Infection, Georgia Institute of Technology, Atlanta, GA 30310

**Keywords:** *Pseudomonas aeruginosa*, cystic fibrosis, *Aspergillus fumigatus*, gliotoxin, polymicrobial interactions

## Abstract

Microbes rarely exist in isolation and instead form complex polymicrobial communities. As a result, microbes have developed intricate offensive and defensive strategies that enhance their fitness in these complex communities. Thus, identifying and understanding the molecular mechanisms controlling polymicrobial interactions is critical for understanding the function of microbial communities. In this study, we show that the gram-negative opportunistic human pathogen *Pseudomonas aeruginosa*, which frequently causes infection alongside a plethora of other microbes including fungi, encodes a genetic network which can detect and defend against gliotoxin, a potent, disulfide-containing antimicrobial produced by the ubiquitous filamentous fungus *Aspergillus fumigatus*. We show that gliotoxin exposure disrupts *P. aeruginosa* zinc homeostasis, leading to transcriptional activation of a gene encoding a previously uncharacterized dithiol oxidase (herein named as DnoP), which detoxifies gliotoxin and structurally related toxins. Despite sharing little homology to the *A. fumigatus* gliotoxin resistance protein (GliT), the enzymatic mechanism of DnoP from *P. aeruginosa* appears to be identical that used by A. fumigatus. Thus, DnoP and its transcriptional induction by low zinc represent a rare example of both convergent evolution of toxin defense and environmental cue sensing across kingdoms. Collectively, these data provide compelling evidence that *P. aeruginosa* has evolved to survive exposure to an *A. fumigatus* disulfide-containing toxin in the natural environment.

## Significance

Bacteria and fungi frequently exist as complex, polymicrobial communities during infection. Reconstructing ecological structure in the laboratory is challenging, and, consequently, the precise molecular mechanisms which underpin microbial interactions remain elusive. Using a preclinical model that mimics the cystic fibrosis lung, we found that the bacterium *Pseudomonas aeruginosa* detects and defends against a disulfide-containing toxin produced by the fungus *Aspergillus fumigatus*. In a remarkable example of both convergent evolution of toxin defense and environmental cue sensing across kingdoms, we found that these organisms use the same cue to produce/sense this toxin and the same enzymatic mechanism to protect against toxicity. This finding provides strong evidence for *P. aeruginosa* exposure to *A. fumigatus*–produced disulfide-containing toxins in natural environments.

Many bacterial infections are not the result of colonization by a single microbe but instead involve polymicrobial communities. Within these communities, coinfecting microorganisms can interact synergistically and/or antagonistically to induce virulence traits, alter the infected niche, increase tolerance to therapeutics, or modulate the host immune response (reviewed in refs. 1–3). Consequently, insight into the mechanisms used by pathogens to interact is key to developing specific agents or strategies to limit infection.

Interactions between filamentous fungi and bacteria are widely studied in the context of human infection, but the precise molecular mechanisms underlying these interactions are understudied. The airways of patients with cystic fibrosis (CF) are inhabited by a functionally diverse polymicrobial community with vast potential for interspecies interactions (1). *Aspergillus fumigatus* and *Pseudomonas aeruginosa* are two of the most prevalent fungal and bacterial pathogens isolated from the CF airway. *A. fumigatus* and *P. aeruginosa* can coinfect the lungs of people with CF, with coinfection ranging from 2 to 45% dependent on the patient cohort (4, 5). In addition, there is correlational clinical evidence that coinfection worsens patient outcomes (4, 6–8), and it has been proposed that interactions between *A. fumigatu*s and *P. aeruginosa* are key to this enhanced pathophysiology. Consequently, there have been numerous in vitro studies examining interactions between *A. fumigatu*s–*P. aeruginosa*, revealing that many of the common *P. aeruginosa* metabolites critical for in vitro interactions with other microbes also impact *A. fumigatus* fitness and physiology during in vitro coculture (9–21). However, much less is known regarding the impact of *A. fumigatus* on *P. aeruginosa* physiology, although recent evidence indicates that *A. fumigatus* soluble factors can alter *P. aeruginosa* physiology (22–25).

Here, we used the CF respiratory environment as a model to unravel mechanistic features of *A. fumigatu*s–*P. aeruginosa* interactions, specifically how *P. aeruginosa* physiology is altered by the presence of *A. fumigatus*. Comparative transcriptomics during growth in a preclinical model mimicking expectorated human CF sputum (26–29) revealed that upon exposure to *A. fumigatus*, *P. aeruginosa* activates a previously uncharacterized gene, PA4170, to levels observed in human CF infection samples. Comparative metabolomics revealed that PA4170 induction during coculture is mediated by zinc starvation, a result of *A. fumigatus* secretion of the natural product gliotoxin. Functional characterization of PA4170 revealed that this gene encodes a disulfide natural product oxidase, which protects *P. aeruginosa* from the redox-cycling effects of gliotoxin. This is the same mechanism which *A. fumigatus* uses to protect itself from self-intoxication with gliotoxin. These data provide evidence of convergent evolution of toxin resistance in *A. fumigatus* and *P. aeruginosa* and suggest that *A. fumigatus* disulfide-containing natural products are ecologically relevant cues sensed by *P. aeruginosa* in natural environments.

## Results

### *P. aeruginosa* Transcriptional Response to *A. fumigatus*.

**Fig. 1.**
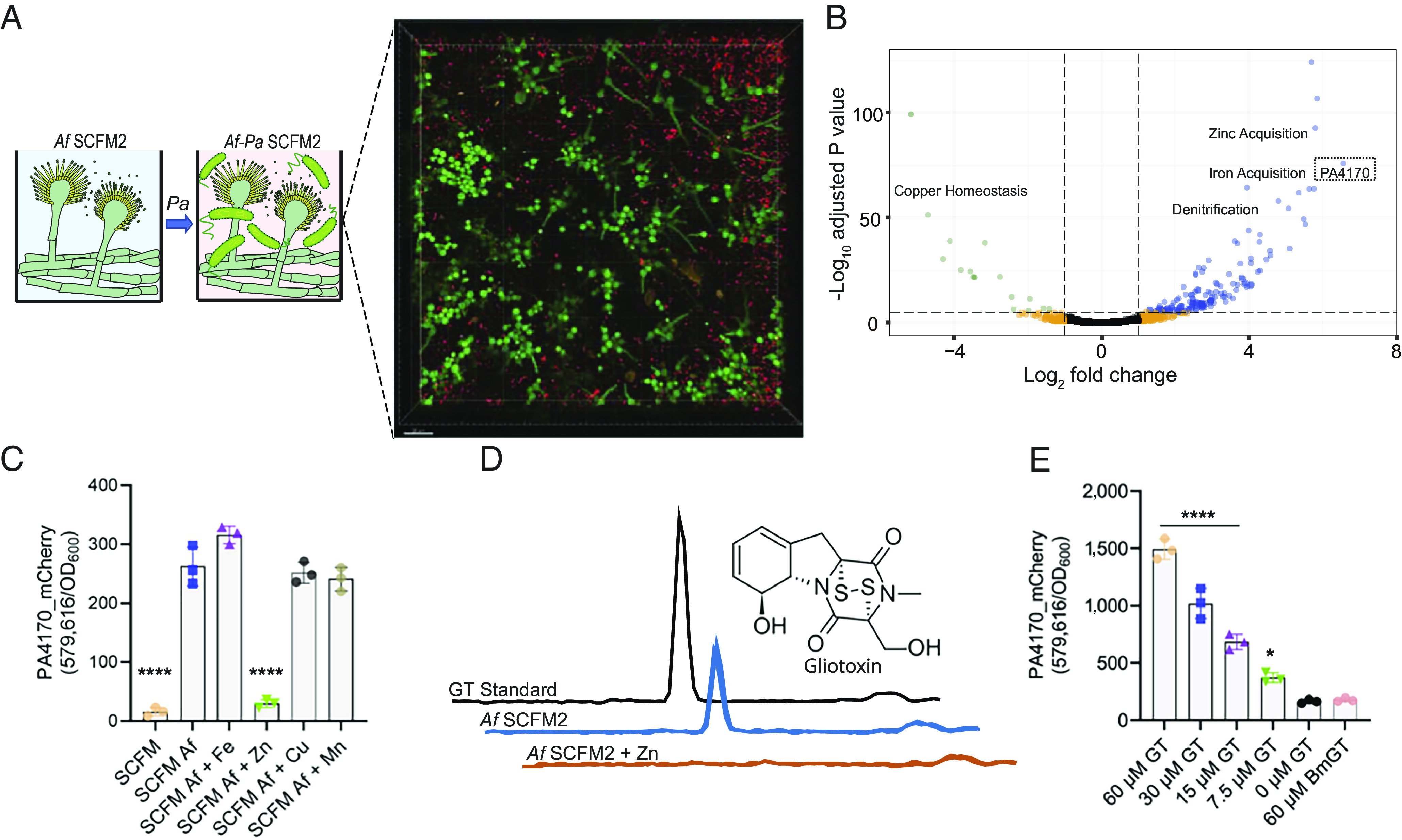
PA4170 is the most highly differentially expressed *P. aeruginosa* gene during coculture with *A. fumigatus* in SCFM2, and expression is activated upon gliotoxin exposure. (*A*) Schematic illustrating the *A. fumigatus* and *P. aeruginosa* coculture setup. *A. fumigatus* is first inoculated into SCFM2 to facilitate germination and hyphal development, followed by *P. aeruginosa* inoculation. Coculture was imaged by confocal laser scanning microscopy (CLSM) at 18 h, 37 °C. *A. fumigatus* was labeled with constitutively expressed mNeonGreen, and *P. aeruginosa* with mCherry. The data are representative of three independent experiments, each performed in triplicate. (Scale bar, 20 µm.) (*B*) Volcano plot illustrating *P. aeruginosa* genes differentially expressed upon coculture with *A. fumigatus*. Bubble colors are used to indicate genes significantly altered in expression with a log_2_ fold change greater than 1 (blue), less than −1 (green), above the adjusted *P*-value threshold (orange), or unchanged (black). Significantly altered clusters of genes (>5) with established functions in *P. aeruginosa* are shown (zinc acquisition, iron acquisition, denitrification, and copper homeostasis). PA4170 (dotted box) was the highest differentially expressed gene (93.7-fold-change). The experiment was performed using biological triplicates. (*C*) mCherry-tagged PA4170 reporter assay examining PA4170 expression following *P. aeruginosa* growth in spent *A. fumigatus* SCFM culture supernatants (24 h) supplemented with different metals (5 µM FeSO_4_, ZnSO_4_, CuSO_4_, and MnSO_4_). Fluorescence was read at 6 h and normalized to cell density (OD_600_). The data are representative of three independent experiments performed in triplicate. Statistical significance was calculated by one-way ANOVA with Dunnett’s multiple comparisons test using “SCFM *Af*” as the control column (****P < 0.0001). Error bars represent SD from the mean. (*D*) Untargeted metabolomics of *A. fumigatus* culture supernatants during growth in SCFM and SCFM + 10 µM ZnSO_4_ (24 h) identified gliotoxin as significantly lowered upon zinc supplementation. Chromatograms show the peak corresponding to gliotoxin (triplicate), alongside a commercial standard. The experiment was performed using biological triplicates. (*E*) mCherry-tagged PA4170 reporter fluorescence assay examining PA4170 protein expression in SCFM following gliotoxin (GT) or bisthiomethylgliotoxin (bmGT) supplementation (0 to 60 µM). Fluorescence was read at 8 h and normalized to cell density (OD_600_). The data are representative of two independent experiments performed in triplicate. Statistical significance was calculated by one-way ANOVA with Dunnett’s multiple comparisons test using “0 µM GT” as the control column (*P = 0.0197 and ****P < 0.0001). Error bars represent SD from the mean.

*A. fumigatus*–*P. aeruginosa* mixed species biofilms form readily in vitro when *A. fumigatus* is inoculated first and allowed to form hyphae, followed by *P. aeruginosa* addition (22, 30). Building on this existing model, we cultured *A. fumigatus* (1 × 10^5^ conidia/mL) statically in synthetic CF medium (SCFM2); a defined medium designed to mimic the chemistry and viscosity of expectorated human CF sputum, which promotes *P. aeruginosa* biofilm aggregate formation (27, 29). After 12 to 16 h, filamentous growth of *A. fumigatus* was observed in SCFM2, at which point, *P. aeruginosa* (5 × 10^5^ cells mL^−1^) was added to the culture. The coculture was allowed to grow until *P. aeruginosa* reached early stationary phase (~12 h, *SI Appendix*, Fig. S1*A*), at which time it was harvested for transcriptome analysis. No significant growth alterations were noted for *P. aeruginosa* or *A. fumigatus* in coculture when compared to monoculture (*SI Appendix*, Fig. S1 *A* and *B*), and there was close association between these microbes during coculture, as shown previously (13, 31, 32) (Fig. 1*A*). This experimental setup allowed us to examine the transcriptional response of *P. aeruginosa* to coculture with *A. fumigatus*.

Upon coculture with *A. fumigatus*, 217 *P. aeruginosa* genes increased and 53 decreased in expression compared to monoculture (fold change ≥ 2 and *P*adj < 0.05, Dataset S1), indicating that there is a robust *P. aeruginosa* response to the presence of *A. fumigatus*. Fig. 1*B* shows an overview of the differentially expressed genes. During growth in oxygen restricted environments, *P. aeruginosa* can anaerobically respire through the process of denitrification, using nitrate as a terminal electron acceptor. Although *A. fumigatus* can grow at low-oxygen concentrations, this fungus cannot grow under strict anaerobic conditions and requires a functional respiratory chain for the initiation of infections (33). Many genes were part of well-characterized regulons involved in adaptation to oxygen levels and micronutrient (iron, copper, and zinc) limitation (Fig. 1*B* and Dataset S1). Genes controlled by the Fnr-type transcription factors Anr and Dnr, which regulate the low-oxygen and denitrification networks in *P. aeruginosa* (34), were up-regulated upon coculture (Dataset S1). This suggests that the competition with *A. fumigatus* for oxygen drives *P. aeruginosa* to utilize alternative electron acceptors. Genes required for synthesis of the iron-scavenging siderophores pyoverdine and pyochelin were also up-regulated (Dataset S1). *P. aeruginosa* central metabolic rewiring in response to iron limitation was also evident upon coculture, with an increased expression of fumarate hydratase (*fumC1*, 27-fold higher) and superoxide dismutase (15-fold higher) (Dataset S1). Genes required for zinc acquisition were also highly up-regulated during coculture (Fig. 1*B* and Dataset S1). This included genes required for synthesis of the zincophore pseudopaline, dedicated zinc uptake systems, and genes encoding zinc-independent ribosomal protein paralogs, which are known to be activated in *P. aeruginosa* upon zinc starvation (35, 36). *dksA2*, encoding a zinc-independent structural analog of the transcription factor DksA (37), was expressed 42-fold higher during coculture, alongside zinc-independent ribosomal subunits. Genes involved in copper homeostasis were highly down-regulated in expression during coculture (Fig. 1*B* and Dataset S1), including c*opA1,* which encodes an ATPase expressed in response to high Cu (38), and the copper-regulated toxin fluopsin C (39). These results indicate that during coculture with *A. fumigatus* in a validated CF preclinical model, *P. aeruginosa* induces genes encoding proteins involved in low-oxygen growth and acquisition of zinc and iron.

### PA4170 Is Activated by a Zinc-Regulated *A. fumigatus* Metabolite.

Although most of the differentially expressed genes upon coculture with *A. fumigatus* could be assigned to known regulons, the top *P. aeruginosa* gene induced (93-fold) upon *A. fumigatus* exposure (Fig. 1*B*), PA4170 (gene designation PA14_09950 in *P. aeruginosa* strain PA14), was uncharacterized. PA4170 is a hypothetical protein, predicted to be encoded on a monocistronic mRNA, and previously shown to be transcriptionally induced during *P. aeruginosa* human chronic infection compared to in vitro monoculture growth (26).

To explore the function of PA4170, we first set out to identify the *A. fumigatus* signal/cue that induces PA4170. To accomplish this, we generated a PA4170-mCherry fusion protein in *P. aeruginosa*, in which mCherry was fused to the C terminus of the full-length protein. This facilitated the quantification of PA4170 production under different growth regimes using a high-throughput fluorescence assay. In agreement with our previous transcriptome results, PA4170 levels were low when *P. aeruginosa* was cultured alone in SCFM2 or standard laboratory media (Fig. 1*C* and *SI Appendix*, Fig. S1*C*). As expected, PA4170 levels increased upon *P. aeruginosa* coculture with *A. fumigatus* in SCFM2 (*SI Appendix*, Fig. S1*C*), and cell-free spent culture supernatants from *A. fumigatus* grown alone also activated PA4170 production (Fig. 1*C*). The PA4170 inducer could be organically extracted from spent *A. fumigatus* culture supernatants with ethyl-acetate, suggesting that it was a nonpolar small molecule (*SI Appendix*, Fig. S1*C*). In addition, spent *A. fumigatus* culture supernatants from as early as 9 to 12 h growth in SCFM2 were capable of inducing *P. aeruginosa* PA4170 expression (*SI Appendix*, Fig. S1*D*).

Since SCFM2 is a low metal environment and *P. aeruginosa* was metal-limited during coculture (Fig. 1*B*), we hypothesized that the PA4170 inducer was synthesized by *A. fumigatus* in response to metal limitation. To test this hypothesis, *A. fumigatus* was grown in SCFM2 in the presence of various metals (iron, zinc, manganese, and copper), and supernatants from these cultures tested for the ability to induce PA4170-mCherry. While supplementation with iron, manganese, and copper had no impact on the ability of *A. fumigatus* supernatants to induce PA4170-mCherry, addition of zinc eliminated induction (Fig. 1*C*). Importantly, the addition of zinc and other metals to *A. fumigatus* culture supernatants obtained after growth in SCFM2 not supplemented with metals retained the ability to activate PA4170 (*SI Appendix*, Fig. S1*E*), indicating that zinc addition does not impact the activity of the PA4170 inducer after synthesis. These data support zinc limitation as a key cue required for *A. fumigatus* synthesis of the PA4170 inducer.

### *A. fumigatus* Gliotoxin Induces PA4170.

To identify *A. fumigatus* metabolites produced during zinc limitation, we performed untargeted metabolomics of *A. fumigatus* culture supernatants following growth in SCFM2 and SCFM2 + zinc. These data revealed that the *A. fumigatus* epipolythiodioxopiperazine (ETP) natural product gliotoxin was produced at high levels in SCFM2 but was undetectable in SCFM2 supplemented with zinc (Fig. 1*D* and *SI Appendix*, Fig. S1*F*). ETPs are a class of disulfide-containing, toxic secondary metabolites made exclusively by fungi (40). Gliotoxin is the most well-studied member of the ETP class and is produced in a prodrug form with an intact disulfide bond (Fig. 1*D*). The disulfide bridge is critical for the intracellular deleterious effects of gliotoxin, mediated through redox-cycling and protein conjugation within target organisms (reviewed in ref. 41). Gliotoxin synthesis is controlled by multiple cues in *A. fumigatus*, including zinc starvation (42, 43), and global fungal secondary metabolism regulators such as LaeA (44). Therefore, it is likely that *A. fumigatus* is zinc-limited during growth in SCFM2, leading to the activation of gliotoxin biosynthesis. In support of this, production of the *A. fumigatus* zinc starvation regulated proteins Aspf2 (45) and GliT (42) was highly expressed during growth in SCFM2 (*SI Appendix*, Fig. S1*G*), and zinc supplementation reduced their synthesis (*SI Appendix*, Fig. S1*G*).

To test whether gliotoxin induces PA4170 expression, *P. aeruginosa* carrying PA4170-mCherry was exposed to gliotoxin, and mCherry levels were quantified. PA4170 was activated by gliotoxin in a dose-dependent manner (Fig. 1*E*). As a control, *P. aeruginosa* carrying PA4170-mCherry was exposed to bisdethiobis(methylthio)gliotoxin (BmGT), a S-thiomethylated, redox-inactive derivative of gliotoxin. BmGT did not activate expression of PA4170 (Fig. 1*E*), indicating that the gliotoxin disulfide bridge gliotoxin was crucial to activate PA4170 expression.

Spent culture supernatants of other *A. fumigatus* strains from clinical (AF293 and AF210) and environmental sources (A1241) were then tested with the *P. aeruginosa* disulfide-containing natural product oxidase in *Pseudomonas* (DnoP)-mCherry reporter assay. All strains substantially induced DnoP expression (*SI Appendix*, Fig. S1*H*). Spent culture supernatants from a Δ*gliZ* mutant (CEA10 background), which cannot synthesize gliotoxin (46), were greatly diminished in their ability induce DnoP expression (*SI Appendix*, Fig. S1*H*). Zinc supplementation prior to *A. fumigatus* inoculation obliterates this response (*SI Appendix*, Fig. S1*I*). These data indicate that gliotoxin is produced by diverse *A. fumigatus* isolates in SCFM2 in response to zinc limitation, and this metabolite induces expression of PA4170 in *P. aeruginosa*.

### Gliotoxin Induces PA4170 Production by Zinc Starvation.

**Fig. 2.**
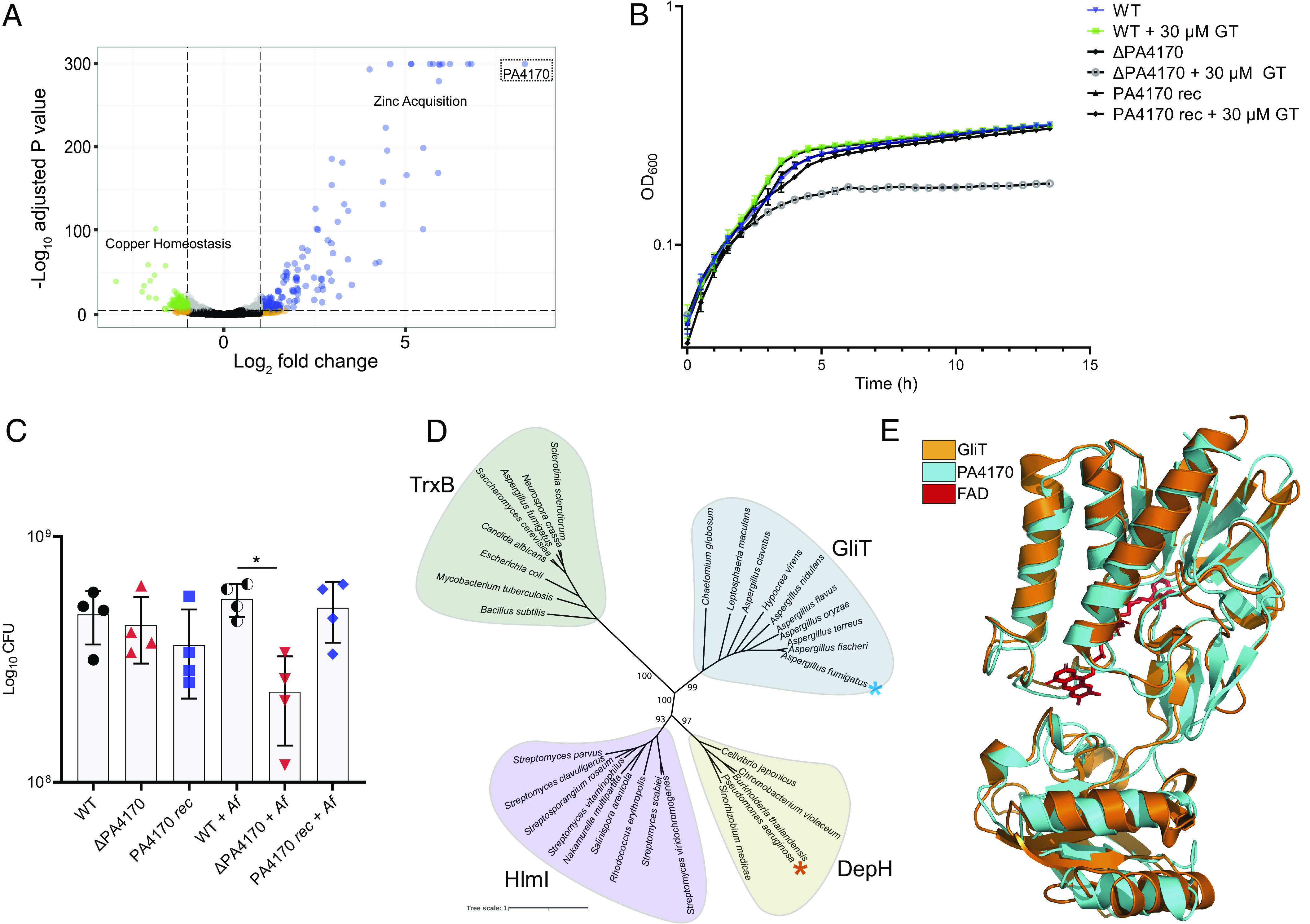
PA4170 encodes a dithiol oxidase which protects *P. aeruginosa* against disulfide-containing natural products. (*A*) Volcano plot illustrating *P. aeruginosa* genes differentially expressed upon exposure to gliotoxin (20 µM) versus a solvent (MeOH) control. Bubble colors are used to indicate genes significantly altered in expression with a log_2_ fold change greater than 1 (blue), less than −1 (green), above the adjusted *P*-value threshold (orange) or unchanged (black/gray). Significantly altered groups of genes (>5) with established functions in *P. aeruginosa* are shown (zinc acquisition and copper homeostasis). PA4170 (dotted box) was the highest differentially expressed gene (313-fold-change). The experiment was performed using biological triplicates. (*B*) Growth of a ΔPA4170 deletion mutant is more susceptible to gliotoxin exposure (30 µM) when compared to the *P. aeruginosa* wild type or PA4170 complemented strain (PA4170 rec). Strains cultured in M9 succinate. An equivalent volume of solvent Dimethylsulfoxide (DMSO) was added to control samples. The data are representative of three independent experiments performed in triplicate. (*C*) Growth of a ΔPA4170 deletion mutant is reduced upon *A. fumigatus* coculture when compared to wild type *P. aeruginosa* during coculture. Growth of ΔPA4170 is not significantly altered compared to the wild type or PA4170 rec (PA4170 plasmid complemented) in monoculture. Cultures were plated for CFU counts 16 h after *P. aeruginosa* addition. CFU = colony-forming unit. The data are representative of three independent experiments performed in quadruplicate. Statistical significance was calculated by the Brown–Forsythe ANOVA test with Dunnett’s T3 multiple comparisons (*P = 0.0215). Error bars represent SD from the mean. (*D*) Phylogenetic analysis of PA4170 alongside representative thioredoxin oxidoreductases (TrxB) and dithiol oxidases from various fungi (GliT) and bacteria (DepH, Hlml). Amino acid sequences were obtained from the GenBank database and aligned with ClustalW. Representative bootstrap values are shown. (*E*) Overlay (PyMOL) of GliT X-ray structure (PDB: 4NTC, orange) with the high-confidence PA4170 AlphaFold (50) predicted structure (Cyan) indicates remarkable structural similarity between these two proteins. Flavin adenine dinucelotide (FAD) was cocrystalized bound in the GliT active site (shown in red).

ETPs are thought to enter cells where it is converted a reduced, active form which can chelate zinc with high affinity (47, 48), thus inhibiting growth. To explore the response of *P. aeruginosa* to gliotoxin in more detail, we carried out an RNA sequencing (RNA-seq) experiment of *P. aeruginosa* exposed to gliotoxin (30 µM) during growth in SCFM2 compared to a solvent control. Gliotoxin exposure resulted in 219 *P. aeruginosa* genes increased and 81 decreased in expression (fold change ≥ 2 and *P*adj < 0.05, Dataset S1). Twenty-nine percent of the genes differentially expressed in the *P. aeruginosa–A. fumigatus* coculture experiment (Fig. 1*A*) were also differentially regulated by gliotoxin, indicating that gliotoxin is an important contributor to the *P. aeruginosa* transcriptome during coculture. Examination of the differentially regulated genes revealed that gliotoxin exposure elicits a specific, zinc starvation response in *P. aeruginosa*, with PA4170 as the most up-regulated transcript (313-fold) (Fig. 2*A*). Several of these up-regulated genes (49) are part of the Zur regulon, which are induced by zinc starvation (Dataset S1), suggesting that PA4170 induction is mediated by zinc starvation. To further test this, *P. aeruginosa* containing the PA4170-mCherry fusion protein was exposed to N,N,N′,N′-tetrakis-(2-Pyridylmethyl) ethylenediamine (TPEN). TPEN is a membrane-permeable, heavy metal chelator with high affinity for zinc. Exposure of *P. aeruginosa* to TPEN activated PA4170 production in a dose-dependent manner, and this activation could be suppressed by zinc supplementation (*SI Appendix*, Fig. S2*A*). These data indicate that ETP-mediated zinc chelation specifically activates PA4170 as part of the *P. aeruginosa* response to zinc depletion, a response downstream of, or parallel to, the Zur regulon.

### PA4170 Plays a Role in *P. aeruginosa* ETP and Dithiolopyrrolone (DTP) Resistance.

To functionally characterize PA4170, a *P. aeruginosa* ΔPA4170 deletion mutant was generated. ΔPA4170 had no discernible growth phenotype during growth in SCFM2 or other laboratory media (Fig. 2*B*). However, this strain was significantly more susceptible to gliotoxin exposure (30 µM) when compared to the wild type (Fig. 2*B*), and this hypersusceptibility could be genetically complemented by introduction of PA4170 into ΔPA4170. ΔPA4170 was not hypersusceptible to bmGT exposure (*SI Appendix*, Fig. S2*B*), or the zinc chelator TPEN (*SI Appendix*, Fig. S2*C*), ruling out a direct role for this protein in *P. aeruginosa* zinc homeostasis. ΔPA4170 also exhibited a growth defect when cultured alongside *A. fumigatus* in SCFM2, compared to wild type *P. aeruginosa* (Fig. 2*C*). This growth defect was not evident when ΔPA4170 was cultured alongside a Δ*gliZ* mutant (*SI Appendix*, Fig. S2*D*). These data indicate that PA4170 is critical for *P. aeruginosa* protection to gliotoxin exposure and fitness during coculture with *A. fumigatus*.

Does PA4170 provide protection against ETPs other than gliotoxin? To answer this question, we exposed wild type *P. aeruginosa* and ΔPA4170 to four additional dithiol-containing microbial-produced toxins, including three ETP toxins (chaetocin, chetomin, and romidepsin) and one DTP toxin (holomycin). DTP toxins are produced by bacteria and similar to ETPs, impact metal homeostasis in both prokaryotic and eukaryotic microbes (47). Growth of ΔPA4170 was reduced upon exposure to chaetocin, chetomin, and holomycin, but not romidepsin, compared to WT *P. aeruginosa* (*SI Appendix*, Fig. S3 *A*–*D*). In addition, exposure to chaetocin, chetomin, and holomycin activated *P. aeruginosa* PA4170 expression (*SI Appendix*, Fig. S3*E*). These data indicate that PA4170 provides protection against a range of microbial-produced dithiol-containing toxins.

### PA4170 Shares Catalytic Motifs with Bacterial and Fungal Natural Product Dithiol Oxidases.

The role of PA4170 in protection against dithiol toxins motivated us to examine the phylogeny of PA4170 in more detail. PA4170 was found in most fully sequenced *P. aeruginosa* strains (279/284), as well as a small number of other pseudomonads including *Pseudomonas fluorescens*, *Pseudomonas chlororaphis*, *Pseudomonas putida*, *Pseudomonas stutzeri,* and *Pseudomonas azotoformans* isolates. Although PA4170 homologs appear to be distributed at random across non–*P. aeruginosa* pseudomonads, it is notably present in all sequenced isolates within a particular *P. chlororaphis* clade (*SI Appendix*, Fig. S4).

An InterPro functional prediction indicated that PA4170 encodes a flavin adenine dinucleotide (FAD) flavoprotein belonging to the family of pyridine nucleotide–disulfide oxidoreductases. These enzymes contain a characteristic pair of redox-active cysteines involved in the transfer of reducing equivalents from the FAD cofactor to a substrate. NCBI Protein Data Bank - Basic Local Alignment Search Tool (PDB-BLAST) was used to scan the Protein Data Bank repository for structurally elucidated, distantly related proteins with sequence similarity to PA4170. Three of the nine proteins identified in this analysis are functionally characterized and were shown to catalyze disulfide bond formation in natural products (*SI Appendix*, Fig. S5 *A*–*C*) including DepH from *Chromobacterium violaceum* (51), HlmI from *Streptomyces clavuligerus* (49), and GliT from *A. fumigatus* (52), which catalyze disulfide bridge closure of romidepsin, holomycin, and gliotoxin respectively.

A phylogenetic analysis of PA4170 and other thioredoxin oxidoreductase (TrxB)-like proteins from various fungi and bacteria revealed a TrxB group, and three separate groups where the structurally elucidated HlmI, GliT, and DepH are representative members (Fig. 2*D*). All enzymes share the conserved CXXC motif which is essential for dithiol oxidase activity (49, 51, 53, 54). The GliT group consists of fungal proteins required for the biosynthesis of ETPs, and it likely diverges from the HlmI and DepH groups because of its fungal origin. PA4170 falls within the DepH group, which also contains TdpH from *Burkholderia thailandensis* E264, known to catalyze disulfide bond formation in thailandepsin A and B biosynthesis (55). While the BLAST analysis indicated a low sequence identity (28.6%) between PA4170 and GliT (*SI Appendix*, Fig. S5*B*), a structural overlay of an AlphaFold generated structural model of PA4170 with the known crystal structure of GliT demonstrated remarkable structural similarity, with conservation at many key motifs and residues known to be required for ETP and DTP oxidation (Fig. 2*E* and *SI Appendix*, Fig. S6). These data suggest that PA4170 protected *P. aeruginosa* against ETP and DTP toxin exposure through disulfide bond regeneration, precisely the same mechanism used by *A. fumigatus* for GliT-mediated self-protection from gliotoxin.

### PA4170 Is a DnoP with Broad Substrate Specificity.

**Fig. 3.**
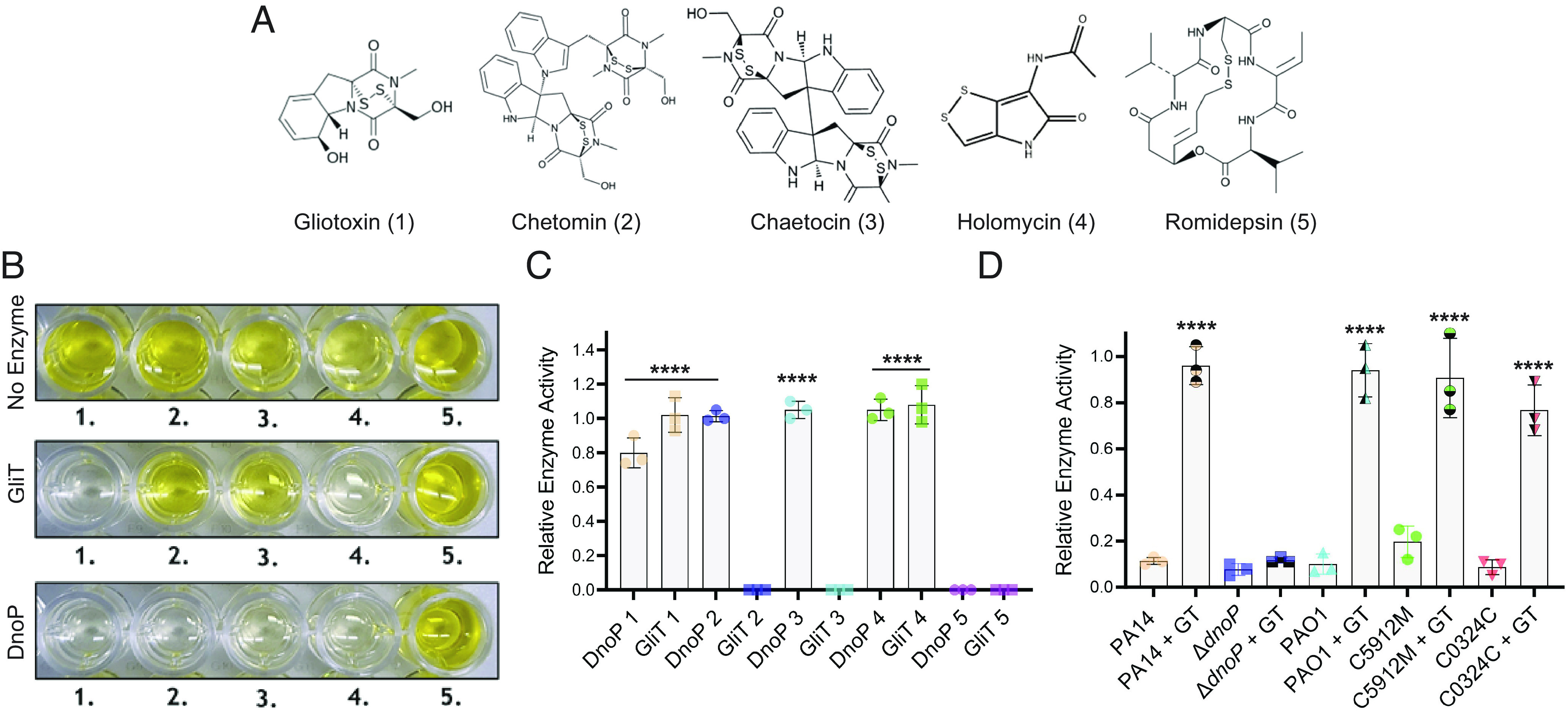
DnoP can catalyze disulfide bond formation for diverse disulfide-containing natural products. (*A*) Structures of the disulfide-containing natural products 1; gliotoxin (*A. fumigatus*), 2; chetomin (*Chaetomium cochliodes*), 3; chaetocin (*Chaetomium minutum*), 4; holomycin (*S. clavuligerus*), and 5; romidepsin (*C. violaceum*). (*B*) 5,5′-dithiobis-(2-nitrobenzoic acid (DTNB) [5,5′-dithio-bis-(2-nitrobenzoic acid)] assay image demonstrating that recombinantly expressed GliT and DnoP can oxidize both reduced gliotoxin (1) and reduced holomycin (4), resulting in the absence of yellow-colored 2-nitro-5-thiobenzoate (TNB) formation upon DTNB addition due to the enzyme-mediated oxidation of the natural product free sulfhydryl groups. DnoP is also capable of oxidizing reduced chetomin (2) and reduced chaetocin (3), whereas neither DnoP nor GliT can oxidize reduced romidepsin (5). Relative enzyme activity (the reduction in TNB formation at 412 nm relative to no-enzyme controls) for DnoP and GliT with the tris(2-carboxyethyl)phosphine (TCEP)-reduced substrates 1 to 5 is shown in (*C*). In each assay, 0.2 µM of DnoP/GliT enzyme was used. Reactions were incubated for 15 min at RT, followed by DTNB addition for 5 min before reading. The data are representative of three independent experiments performed in triplicate. The third replicate was performed with 0.2 µM GliT-mCherry and DnoP-mCherry purified from *A. fumigatus* (*SI Appendix*, Fig. S7*B*). Statistical significance was calculated by one-way ANOVA with Dunnett’s multiple comparisons test using no enzyme added as the control (****P < 0.0001). Error bars represent SD from the mean. (*D*) Relative DnoP enzyme activity (the reduction in TNB formation at 412 nm relative to no-enzyme controls) of *P. aeruginosa* whole cell lysates from PA14, Δ*dnoP*, PAO1, C5912M, and C0324C, using TCEP-reduced gliotoxin as a substrate. DnoP enzyme expression was induced through the addition of gliotoxin (20 µM) and compared to the addition of a solvent control (MeOH). Reactions were incubated for 15 min at RT, followed by DTNB addition for 5 min before reading. The data are representative of three independent experiments performed in triplicate. Statistical significance was calculated by one-way ANOVA with Dunnett’s multiple comparisons test using “PA14” as the control column (****P < 0.0001). Error bars represent SD from the mean.

Intrigued by the ability of this single enzyme to protect *P. aerugino*sa against multiple disulfide-containing natural products (*SI Appendix*, Fig. S3 *A*–*D*), we investigated the enzymology of PA4170 in more detail. Both GliT and PA4170 were recombinantly expressed in *Escherichia coli*, purified to homogeneity, and assessed for their ability to oxidize the ETP/DTP toxins gliotoxin, chaetocin, chetomin, romidepsin, and holomycin. Recombinant PA4170 and GliT catalyzed disulfide oxidation of both gliotoxin and holomycin (Fig. 3 *A* and *B*). However, PA4170 was also capable of restoring the disulfide bridge of the reduced ETPs chetomin and chaetocin, whereas GliT was unable to act on these metabolites. Neither enzyme was capable of oxidizing reduced romidepsin (Fig. 3 *A*–*C*). Based on these data, we herein assigned *P. aeruginosa* PA4170 as DnoP. From examining the bulk *P. aeruginosa* transcriptome from CF patients colonized with this microbe across different CF clinics, we uncovered that the *dnoP* transcript is expressed highly during human infection (19, 20, 23). However, *P. aeruginosa* chronic infection isolates are highly diverse, so although expression of the *dnoP* transcript was detected, these individual isolates may not make a functional DnoP protein. To address this question, we modified our dithiol oxidase enzyme assay to test the DnoP activity within *P. aeruginosa* cell lysates. Diverse *P. aeruginosa* isolates (PA14, PA14 Δ*dnoP*, PAO1, C5912M, and C2773C) were treated with gliotoxin (or a solvent control) during growth in SCFM, and the resulting cell lysates were tested for DnoP activity. As shown in Fig. 3*D*, inducible DnoP activity was readily detected in diverse *P. aeruginosa* isolates. C5912M is a mucoid *P. aeruginosa* isolate from a patient with CF (56). This result suggests that mucoidy, which is known to impact antimicrobial efficacy (33), does not impact the ability of gliotoxin to activate *dnoP* expression. Importantly, gliotoxin exposed cell lysates from the PA14 Δ*dnoP* mutant had only background levels DnoP enzyme activity. These data conclusively show that DnoP functionality is conserved across *P. aeruginosa* isolates.

### DnoP Functionally Complements *A. fumigatus* Δ*gliT*.

**Fig. 4.**
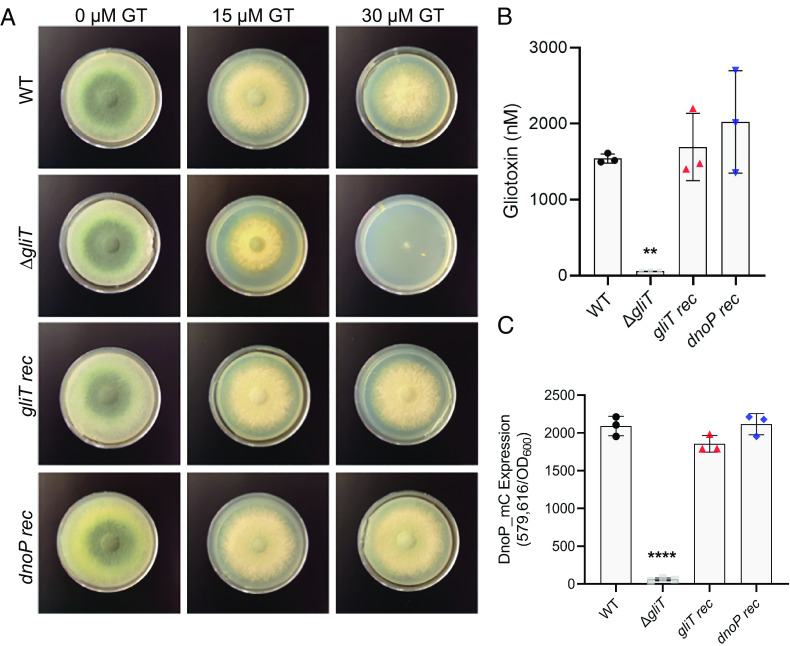
Heterologous expression of *dnoP* in *A. fumigatus* can functionally complement for the loss of the dithiol gliotoxin oxidase *gliT*. (*A*) Plate assay of *A. fumigatus* wild type, Δ*gliT*, *gliT rec,* and *dnoP rec* growth upon gliotoxin addition (0, 15, and 30 µM) to GMM agar plates. Plates imaged at 48 h. The data are representative of three independent experiments performed in triplicate. (*B*) Complementation of Δ*gliT* in a neutral safe-haven site with *gliT* (*gliT rec*) or *dnoP* (*dnoP rec*) restores *A. fumigatus* gliotoxin production to wild type levels. Gliotoxin was quantified using LC–MS following 72 h growth in SCFM2. The experiment was performed in triplicate. Statistical significance was calculated by one-way ANOVA with Dunnett’s multiple comparisons test using “WT” as the control column (**P = 0.0052). Error bars represent SD from the mean. (*C*) Complementation of Δ*gliT* in a neutral site with *gliT* (*gliT rec*) or *dnoP* (*dnoP rec*) restores the ability of *A. fumigatus* spent culture supernatants (24 h) to activate *P. aeruginosa* PA4170 expression. Fluorescence was read at 10 h and normalized to cell density (OD_600_). The data are representative of two independent experiments performed in triplicate. Statistical significance was calculated by one-way ANOVA with Dunnett’s multiple comparisons test using WT as the control column (****P < 0.0001). Error bars represent SD from the mean.

At this point, it was unclear whether the broader substrate specificity of DnoP results in a significant cost to catalytic function. To explore this, we tested the ability of heterologously expressed DnoP to substitute for the combined biosynthetic and self-defense functionalities of GliT in *A. fumigatus*. An *A. fumigatus* Δ*gliT* mutant was generated, which does not produce gliotoxin due to an inability to catalyze the final disulfide bridge closure. This mutant is also susceptible to exogenous gliotoxin exposure (52). Using CRISPR-Cas9, *A. fumigatus* Δ*gliT* was genetically complemented by placement of a DNA fragment consisting of the *gliT* native promoter and the *gliT* open reading frame (ORF) with an in-frame C-terminal mCherry tag (*gliT*_mC) into an intergenic “safe haven” region of the *A. fumigatus* genome. The same procedure was used create another strain in which a DNA fragment containing the *P. aeruginosa* DnoP ORF with a C-terminal mCherry tag (*dnoP*_mC), driven by the *gliT* promoter, was inserted into *A. fumigatus* Δ*gliT*. As expected, complementation of Δ*gliT* with *gliT*_mC restored self-protection against gliotoxin to wild type levels (Fig. 4*A*). Complementation of Δ*gliT* with *dnoP*_mC also restored gliotoxin self-protection, in a manner indistinguishable to the native enzyme (Fig. 4*A*). Furthermore, both *gliT_mc* and *dnoP_mc* complemented Δ*gliT* had gliotoxin biosynthesis fully restored upon growth in SCFM2 (Fig. 4*B*) and spent culture supernatants from these complemented strains activated DnoP expression in *P. aeruginosa* (Fig. 4*C*). Finally, purified GliT-mC and DnoP-mC (*SI Appendix*, Fig. S7*B*) exhibited identical substrate specificity to the His-tagged versions of these proteins recombinantly expressed in *E. coli* (Fig. 3*C*). These data indicate that DnoP can effectively substitute for *A. fumigatus* GliT functionality, despite its low protein sequence identity and broad substrate specificity.

## Discussion

Many microbes exist in natural environments as complex, interkingdom polymicrobial communities. Consequently, microbes have evolved diverse mechanisms to compete with their neighbors for space and resources. Nutritional resources are at the forefront of microbial competition, and microbes have evolved numerous strategies to increase nutrient acquisition or to actively restrict them from others (57, 58). We have identified and characterized a *P. aeruginosa* DnoP engaged in xenobiotic defense against a family of potent small molecule toxins that function by zinc sequestration. Importantly, DnoP was uncovered in *P. aeruginosa*, an organism which does not synthesize disulfide-containing natural products. All other dithiol oxidases characterized to date are encoded within natural product biosynthetic gene clusters, dedicated primarily to the synthesis of these toxins. Although the natural substrate of DnoP is uncertain, we uncovered that *dnoP* expression is activated upon coculture with *A. fumigatus*, and that a Δ*dnoP* mutant has a fitness defect during coculture with this ubiquitous, saprophytic fungus. Overall, the widespread conservation of *dnoP* in diverse *P. aeruginosa* isolates suggests that this bacterium frequently encounters ETP-producing filamentous fungi or DTP-producing bacteria in nature and has evolved a single protein system to defend against their toxins. It is likely that the broader role of ETPs and other dithiol-containing natural products in microbial interactions has been masked by the presence of cognate resistance genes like *dnoP*.

Microbial stress responses which detect ecological competition, such as nutrient limitation, are often coupled with the release of toxins, as this form of competition normally implies the presence of foreign genotypes. Nevertheless, it is unclear why the limitation of one nutrient but not another should promote toxin production (59). We propose that, as ETPs dysregulate zinc homeostasis in target organisms, orchestrating gliotoxin production via zinc starvation-responsive transcriptional regulators allows *A. fumigatus* to synthesize this toxin when it will have maximum impact on target organisms. The efficacy of an intracellular zincophore is likely to have maximum impact on target cells when the capacity of these competitors to obtain zinc is limited. Alternatively, it may be preferential for *A. fumigatus* to synthesize gliotoxin when its metabolism is transcriptionally wired to endure zinc limitation, as microbes are acutely sensitive to shifts in cellular free zinc levels. It is likely that ETP biosynthesis requires a careful coordination between primary and secondary metabolism to avoid self-intoxication. Orchestrating ETP biosynthesis through a zinc starvation response may also facilitate cooperation in neighboring ETP-producing fungi, catalyzing a positive feedback loop of toxin production (60). This fundamental regulatory cue may be conserved across multiple dithiol toxin-producing organisms. Indeed, zinc starvation was shown to activate holomycin production in the gram-negative marine bacterium *Photobacterium galatheae* (61).

Stress responses are an effective cue to provide a finely tuned, graded readout of ecological competition and its capacity to reduce cellular fitness. One which is receptive to both alterations in community species composition and to variations in the chemical signatures of competitors (59). In an example of cross-kingdom cue convergence, we have shown that *P. aeruginosa* expresses DnoP in response to zinc starvation. The precise signaling cascade leading to *dnoP* activation has yet to be elucidated. However, this method of transcriptional regulation may provide *P. aeruginosa* preemptive protection against ETP exposure during growth in zinc-limited environments, where gliotoxin production by *A. fumigatus* is maximal. Alternatively, the activation of *dnoP* by zinc starvation may allow for sustained *dnoP* expression upon ETP-mediated disruption of cellular zinc homeostasis, facilitating survival. Thus, zinc limitation may be a conserved signal across kingdoms for both dithiol-containing natural product production and defense.

The ability of *dnoP* to fully complement for *gliT* functionality in *A. fumigatus* was unexpected. This suggests that there is minimal catalytic tradeoff in retaining a broader substrate range with these enzymes. Specialization is driven by requirements for adequate regulation and localization, as well as for high catalytic efficiency with the target substrate (62). The substrate specialization commonly found in dedicated natural product dithiol oxidases implies that the broad substrate specificity of DnoP is by design. It will be interesting to further explore the substrate specificity of DnoP from other organisms to see whether this is a pervasive trend. In addition to providing evolutionary insight, these promiscuous enzymes may yield useful starting material for protein engineering and synthetic biology approaches.

## Materials and Methods

### Media and Growth Conditions.

*P. aeruginosa* cells were routinely cultured in lysogeny broth (LB LENNOX) at 37 °C with shaking (250 rpm) unless otherwise specified. All liquid culture experiments were performed in M9-succinate or SCFM unless explicitly stated otherwise. *Pseudomonas* isolation agar (PIA) was used for selecting *P. aeruginosa* against *A. fumigatus* for CFU plating. *Escherichia coli* cells were cultured in LB. To prepare plates, 1.5% (wt/vol) agar was used. Antibiotics were added to the media as necessary: 60 µg mL^−1^ of gentamicin (Gen), 75 µg mL^−1^ s tetracycline (Tet), or 300 µg mL^−1^ carbenicillin (Cb) for *P. aeruginosa*; 10 µg mL^−1^ of Gen, 10 µg mL^−1^ Tet, or 100 µg mL^−1^ Cb for *E. coli*. Cell growth was monitored as optical density in a spectrophotometer at a wavelength of 600 nm (OD_600_).

*A. fumigatus* and other *Aspergillus* isolates were stored as conidia in 25% glycerol at −80 °C and maintained on 1% glucose minimal medium (GMM). Solid medium was prepared by addition of 1.5% agar. All experiments were performed with GMM, SDB (Sabouraud-dextrose broth), or SCFM unless explicitly stated otherwise. For all experiments, *A. fumigatus* was grown on solid GMM at 37 °C for 3 d to produce conidia. Conidia were collected using 0.01% Tween 80, counted using a hemocytometer, and diluted in medium to the final concentration used in each assay.

### *A. fumigatus* and *P. aeruginosa* SCFM Coculture Model.

*A. fumigatus* was incubated statically at 37 °C in 5 mL SCFM2 (10^5^ conidia/mL), in 6-well flat bottom cell culture plates until germination and hyphal development (18 h). *P. aeruginosa* was precultured in LB overnight; then, 50 μL of the culture was inoculated into 5 mL of SCFM and incubated the culture at 37 °C for 6 h until the cultures reached mid-log phase (optical density at 600 nm [OD_600_] = 0.5). The OD_600_ was adjusted to 0.001 in 5 mL of SCFM2, and this 5 mL was added to the germinated *A. fumigatus* culture to establish the coculture, or into 5 mL SCFM2 for *P. aeruginosa* monoculture [*P. aeruginosa* starting OD_600_ = 0.0005 (~5 × 10^5^ cells mL^−1^)]. *P. aeruginosa* CFU was tracked over time by plating on PIA. Cultures were grown until *P. aeruginosa* reached stationary phase (12 to 14 h). *A. fumigatus* mycelial wet weight (mg/mL^−1^) was quantified by filtering cultures containing *A. fumigatus* through a 40 µm cell strain and rising with PBS. Coculture biofilm images were acquired with a Zeiss LSM 880 confocal laser scanning microscopy utilizing Zen image capture software. Detection of mCherry-expressing *P. aeruginosa* cells was performed with an excitation wavelength centered at 587 nm and an emission wavelength centered at 610 nm. Detection of mNeonGreen-expressing *A. fumigatus* was performed using an excitation wavelength centered at 488 nm and an emission wavelength centered at 509 nm. All images were acquired using a 63× oil-immersion objective.

### *P. aeruginosa* and *A. fumigatus* Coculture RNA-seq.

Whole cultures (biofilm and planktonic cells) of *P. aeruginosa* monocultures and cocultures with *A. fumigatus* were harvested in the stationary phase of growth (triplicates) by aspirating the contents of the petri-dish well into a 15 mL falcon tube (passed over a 40 µm cell strainer) and combining this with an equal volume of RNA-later. Cultures stored in RNA-later were pelleted, resuspended in 1 mL RNA Bee, and RNA was extracted as described previously. Sequencing was performed by the Microbial Genome Sequencing Center (https://www.migscenter.com/). Default parameters were used for all software. Trimmed reads were then mapped to *P. aeruginosa* PA14 reference (available for download from Pseudomonas.com) genome using Bowtie2 v2.4.2 with default parameters for end-to-end alignment. Read summarization was performed using featureCounts (63). DESeq2 was employed to analyze differentially expressed genes (64). Annotations of differentially expressed genes were obtained from the reference annotation of the *Pseudomonas* genome available at the Pseudomonas Genome Database (PGD) website. Genes were considered significantly altered when their adjusted *P*-value was <0.05 (Dataset S1). The sequencing data are deposited at ArrayExpress (accession number E-MTAB-12786).

### *P. aeruginosa* GT Exposure RNA-seq.

*P. aeruginosa* PA14 was precultured as described above, and the OD_600_ was adjusted to 0.05 in 8 mL of SCFM2, in 6-well flat bottom cell culture plates. Gliotoxin (20 µM) or MeOH (solvent control) was added at log phase (optical density at 600 nm [OD_600_] = 0.3), and the cultures were incubated statically for an additional 4 h. Then, 5 mL of each culture (triplicate) was immediately added to an equal volume of RNA-later. RNA isolation, sequencing, and mapping were carried out as described above. The sequencing data are deposited at ArrayExpress (accession number E-MTAB-12785).

### Statistics.

All experiments were performed independently at least three times (unless otherwise stated) with similar observations. The exact *n* values for experiments are provided in figures and legends. Statistical analyses were conducted using Prism GraphPad 10 and specified in figure legends. Unless otherwise noted, all experiments were performed with a minimum of three biologically independent samples.

### PA4710-mCherry Reporter Assay.

The *P. aeruginosa* PA4170-mCherry fusion protein reporter strain was subcultured to an OD_600_ of 0.05 in SCFM, and 20 µL was then added to 96-well plate wells containing *A. fumigatus* spent culture supernatants or SCFM containing the indicated supplements (180 µL). The resulting plates were incubated statically at 37 °C for the indicated times (6 to 12 h). Optical density (OD_600_) and fluorescence were measured in a BioTek SynergyMx plate reader (excitation, 587 nm; emission, 620 nm) with Gen5 software.

### *A. fumigatus* mCherry Reporter Assays.

*A. fumigatus* mCherry fusion protein strains were inoculated into SCFM (10^5^ conidia/mL to 10 mL) into 6-well flat bottom cell culture plates. Plates were incubated statically at 37 °C for 48 h. Fluorescence was measured in a BioTek SynergyMx plate reader (excitation, 587 nm; emission, 620 nm) with Gen5 software. GliT-mCherry and Aspf2-mCherry fluorescence was normalized to *gpdA*p grown in identical culture conditions to account for any growth variations.

### *A. fumigatus* Gliotoxin Exposure Plate Assays.

Radial growth assays were performed by inoculating GMM agar plates containing the indicated quantity of gliotoxin with 10^3^ conidia in 5 μL 0.01% Tween 20, followed by incubation at 37 °C for 48 h and imaging.

### LC–MS Analysis of Gliotoxin.

Quantification of gliotoxin production was carried out by liquid chromatography–mass spectrometry (LC–MS) as described previously (65). Briefly, culture supernatants from *A. fumigatus* grown for the indicated duration (24 to 72 h) in SCFM2 were harvested and stored at −20 °C. Then, 100 µL of samples was added into a new tube, and 200 µL of ice-cold MeOH: ACN:H_2_O (5:3:2/V:V:V) was added. Samples were vortexed twice, centrifuged at 21,100× *g* for 5 min, and then, 100 µL of the sample was added to an LC vial. Ultraperformance liquid chromatography–mass spectrometry was performed using an UltiMate 3000 fitted with a Waters AcquityUPLC BEH C18 column (100 × 2.1 mm, 1.7 μ) coupled to Orbitrap ID-X mass spectrometry system.

The chromatographic method for sample analysis involved elution with H_2_O and 0.1% formic acid (mobile phase A) acetonitrile (ACN) and 0.1% formic acid (mobile phase B) at 0.4 mL min^−1^ flow rate using the following gradient program: 0 min 5% B; 4 min 30% B; 7 min 100% B; hold to 8.9 min, and then 9 min 5% B hold to 10 min. The column temperature was set to 50 °C, and the injection volume was 5 µL. The targeted molecule gliotoxin fragmented by HCD at 35 collision energy in positive mode. MS/MS transitions for gliotoxin are 327.05/263.08. Standard curves of gliotoxin were generated. The amount of gliotoxin in the samples was calculated based on the standard curve.

### Phylogenetic Analysis of PA4170.

Nucleotide BLAST (BLASTN) and protein BLAST (BLASTP) searches were performed on Pseudomonas.com and NCBI to identify orthologs of PA4170 across the *Pseudomonas* genus and other organisms. DNA sequences encompassing the PA4170 locus from the *P. aeruginosa* PA14 strain were used as the query. Orthologs identified from complete bacterial genomes with E values of <1 × 10^−4^, percent identity >90%, and query coverage values of >90% were retained for further analysis. Sequences of PA4170 orthologs were aligned using ClustalW. The alignments were visualized in Jalview (66) using the default nucleotide color scheme. For the phylogenetic analysis of PA4170 alongside representative TrxB and dithiol oxidases from various fungi (GliT) and bacteria (DepH, Hlml). Amino acid sequences were obtained from the GenBank database and aligned with ClustalW. Phylogenetic analysis was performed using maximum likelihood methods (MEGA11). Representative bootstrap values were calculated and the tree was visualized and annotated in Interactive Tree Of Life (iTOL) (67). A maximum-likelihood phylogenetic tree for *Pseudomonas* was constructed in MEGA (68) using 358 16 s rRNA sequences representing diverse isolates. For better visualization of the tree, we only included approximately 50 16 s sequences from the *P. aeruginosa* strains. The 16 s sequences were aligned with ClustalW and used as an input in MEGA11. The tree was visualized and annotated in iTOL (67).

### Plasmids and Strains.

Strains and plasmids used in this study are listed in *SI Appendix*, Table S1. All plasmids were verified by PCR, restriction enzyme digestion, and if necessary, DNA sequencing. Verified plasmids for targeting *P. aeruginosa* were transformed into *E. coli* SM10 λ*pir*, followed by conjugation into *P. aeruginosa* PA14 as described previously. Appropriate antibiotics were used for selection as indicated. Fungal strains outside those generated in this study were obtained from the Fungal Genetics Stock Center (Manhattan, Kansas, USA).

### *A. fumigatus* Genetic Manipulation.

*A. fumigatus* strain A1160 *Δku80 pyrG*+ was used as the genetic background for the deletion of *gliT and gliZ*. Both mutants were generated by replacing the ORF with the dominant selection marker *hygB* (hygromycin resistance). The replacement construct was generated using overlap PCR to fuse ∼1 kb upstream and ∼1 kb downstream of the open reading frame of *gliT/gliZ* to the *hygB* marker. The resulting constructs were transformed into protoplasts of each strain, and mutants were selected on osmotically stabilized minimal medium (GMM plus 1.2 M sorbitol) containing 200 µg/mL hygromycin B (Sigma). Protoplasts were generated using lysing enzyme and transformed as previously described. Strains were single spored and checked for correct integration, or the presence of construct in the case of the ectopic reconstituted strains via PCR.

Transformation of *A. fumigatus* with *gliT*-mCherry, *dnoP*-mCherry, *gpdA*p-mCherry, or *aspf2*-mCherry into the *A. fumigatus* SH1 neutral domain was carried out using a CRISPR/Cas9-mediated protocol for gene editing, as previously described (69). crRNA was purchased from Integrated DNA Technologies, Inc. Complete guideRNAs (gRNAs) were then assembled in vitro using the custom-designed crRNA coupled with a commercially acquired tracrRNA. The assembled gRNAs were then combined with commercially purchased Cas9 to form ribonucleoproteins for transformation, as previously. Repair templates carrying a phleomycin resistance (*bleoR*) cassette were PCR amplified to contain 40-basepair regions of microhomology on either side for homologous integration at the double-strand DNA break induced by the Cas9 nuclease. Protoplast-mediated transformations were then carried out using the hygromycin repair templates and Cas-ribonucleoproteins for gene targeting. Homologous integrations were confirmed by PCR.

### GliT and DnoP Protein Expression and Purification.

The PCR-amplified ORFs of *gliT* and *dnoP* were cloned into the expression vector pET-19 m, which introduces a TEV-cleavable N-terminal hexahistidine tag onto each protein. For purification of the His_6_-tagged proteins, the cells were grown in Turbo Broth medium (Molecular Dimensions) (1 L) at 37 °C with good aeration to *A*_600_ = 0.5. The temperature was then lowered to 16 °C, and isopropyl 1-thio-β-D-galactopyranoside was added to 1 mM final concentration to induce expression of the cloned genes. The induced cultures were grown for a further 16 h and then harvested by centrifugation (6,000× *g*, 4 °C, 15 min). The cell pellet was resuspended in 20 mL of buffer A [50 mM sodium phosphate, 100 mM NaCl, and 10% (v/v) glycerol, pH 8.0, containing one dissolved cOmplete™ EDTA-free Protease Inhibitor cocktail tablet (Roche)]. The cell lysate was clarified by centrifugation (11,000× *g*, 4 °C, 30 min), and the filtered lysate was then loaded onto a Ni-NTA spin column (Qiagen). The column was washed five times with buffer A containing 10 mM imidazole. The His_6_-tagged proteins were eluted with buffer A containing 250 mM imidazole. Protein was concentrated and buffer exchanged to the desired concentration using an Amicon® Ultra-4 Centrifugal Filter (10 kDa NMW cutoff).

For GliT-mCherry and DnoP-mCherry expression and purification from *A. fumigatus*, 100 mL (10^5^ conidia/mL) of *A. fumigatus* strains *gliT* rec and *dnoP* rec were cultured in SDB for 24 h, followed by 10 µM gliotoxin addition for 6 h. Mycelia was then harvested, flash-frozen in liquid nitrogen, and ground to a fine powder using a pestle and mortar. Ground mycelia was resuspended in 20 mL of buffer A and transferred to bead-beating tubes. Cells were lysed by bead beating twice for 20 s, and the tubes were placed on ice for 1 min between each homogenization. The cell lysates were clarified by centrifugation (11,000× *g*, 4 °C, 30 min), and the filtered lysates were added to an Eppendorf containing 200 µL of prewashed ALFA Selector PE magnetic beads (NanoTag Biotechnologies). Samples (4 mL) were then placed on an end-over-end orbital rotator for 1 h at 4 °C, placed on a magnetic stand, and the beads were washed five times with 1 mL of buffer A, followed by elution with 200 µL buffer A + 200 µM ALFA elution peptide. Proteins were inspected by Sodium Dodecyl Sulphate-Polyacrylamide Gel Electrophoresis (SDS-PAGE) analysis for purity.

### Dithiol Oxidase Enzyme Assay.

The dithiol oxidase activity of GliT and DnoP was measured based a method described previously (70). Briefly, the free thiol groups on reduced disulfide natural products react with 5,5′-dithio*bis*(2-nitrobenzoic acid) (DTNB) to yield 2-nitro-5-thiobenzoate (TNB^2−^) anions. TNB^2−^ is colored and its formation can be monitored at 412 nm. The reaction mixtures (180 µL) contained buffer (20 mM Tris-HCl, pH 7.0, and 100 mM NaCl) and TCEP-reduced substrates (gliotoxin, holomycin, chetomin, chaetocin, and romidepsin) at 40 µM final. The reaction was initiated by the addition of GliT or PA4170 (to a final concentration of 0.2 µM) and incubated at room temperature for 15 min. DTNB dissolved in EtOH (5 mM) was used as a color development substrate after the oxidase reaction was complete. Enzyme-free controls were used to monitor background natural product reoxidation, which was not significant over the course of the assay timeframe. The A_412_ was measured in a BioTek SynergyMx.

### Gliotoxin Oxidase Enzyme Assay Using *P. aeruginosa* Cell Lysates.

Testing the gliotoxin oxidase activity of *P. aeruginosa* cell lysates was carried out through modifications of the above dithiol oxidase enzyme assay. Briefly. *P. aeruginosa* strains were cultured in 30 mL SCFM (static, 37C) until the cultures reached log phase (optical density at 600 nm [OD_600_] = 0.3). Gliotoxin (20 µM) or MeOH (solvent control) was then added to the cultures, which were incubated for a further 6 h. Cells from the above cultures were harvested by centrifugation (8,000× *g*, 4 °C, 15 min). The cell pellet was resuspended in chilled lysis buffer (BugBuster) containing one dissolved cOmplete™ EDTA-free Protease Inhibitor cocktail tablet (Roche). The cell lysates were clarified by centrifugation (11,000× *g*, 4 °C, 30 min), and the final cell lysate protein content was quantified by Bicinchoninic acid (BCA) assay (Pierce™ BCA Protein Assay). Cell lysates were normalized to 10 mg/mL, and the enzyme assay was carried out immediately using fresh lysates. Reaction mixtures (200 µL) contained buffer (20 mM Tris-HCl, pH 7.0, and 100 mM NaCl) and TCEP-reduced gliotoxin at 40 µM final. The reaction was initiated by the addition of *P. aeruginosa* cell lysate (to a final concentration of 50 µg per reaction), and mixtures were incubated at room temperature for 15 min. DTNB dissolved in EtOH (5 mM) was used as a color development substrate after the oxidase reaction was complete. The A_412_ was measured in a BioTek Synergy H1 Hybrid Reader.

## Supplementary Material

Appendix 01 (PDF)

Dataset S01 (XLSX)

## Data Availability

The raw sequencing files from this study are available at the functional genomics data collection (ArrayExpress) under accession numbers E-MTAB-12786 and E-MTAB-12785. All study data are included in the article and/or supporting information.

## References

[1] C. B. Ibberson, M. Whiteley, The social life of microbes in chronic infection. Curr. Opin. Microbiol. **53**, 44–50 (2020). 32145635 10.1016/j.mib.2020.02.003 PMC7244389

[2] W. Little, C. Black, A. C. Smith, Clinical implications of polymicrobial synergism effects on antimicrobial susceptibility. Pathogens **10**, 144 (2021). 33535562 10.3390/pathogens10020144 PMC7912749

[3] J. L. Murray, J. L. Connell, A. Stacy, K. H. Turner, M. Whiteley, Mechanisms of synergy in polymicrobial infections. J. Microbiol. **52**, 188–199 (2014). 24585050 10.1007/s12275-014-4067-3 PMC7090983

[4] E. Reece , Co-colonisation with Aspergillus fumigatus and Pseudomonas aeruginosa is associated with poorer health in cystic fibrosis patients: An Irish registry analysis. BMC Pulm. Med. **17**, 70 (2017). 28431569 10.1186/s12890-017-0416-4 PMC5401475

[5] J. Zhao, W. Cheng, X. He, Y. Liu, The co-colonization prevalence of Pseudomonas aeruginosa and Aspergillus fumigatus in cystic fibrosis: A systematic review and meta-analysis. Microb. Pathog. **125**, 122–128 (2018). 30217514 10.1016/j.micpath.2018.09.010

[6] R. Amin, A. Dupuis, S. D. Aaron, F. Ratjen, The effect of chronic infection with Aspergillus fumigatus on lung function and hospitalization in patients with cystic fibrosis. Chest **137**, 171–176 (2010). 19567494 10.1378/chest.09-1103

[7] D. A. Hughes , Clinical characteristics of Pseudomonas and Aspergillus co-infected cystic fibrosis patients: A UK registry study. J. Cyst. Fibros. **21**, 129–135 (2022). 33958279 10.1016/j.jcf.2021.04.007

[8] D. Shoseyov, K. G. Brownlee, S. P. Conway, E. Kerem, Aspergillus bronchitis in cystic fibrosis. Chest **130**, 222–226 (2006). 16840406 10.1378/chest.130.1.222

[9] R. Anand, K. V. Clemons, D. A. Stevens, Effect of anaerobiasis or hypoxia on Pseudomonas aeruginosa inhibition of Aspergillus fumigatus biofilm. Arch. Microbiol. **199**, 881–890 (2017). 28357473 10.1007/s00203-017-1362-5

[10] R. Anand , Small colony variants of Pseudomonas aeruginosa display heterogeneity in inhibiting Aspergillus fumigatus biofilm. Mycopathologia **183**, 263–272 (2018). 28785939 10.1007/s11046-017-0186-9

[11] B. Briard , Pseudomonas aeruginosa manipulates redox and iron homeostasis of its microbiota partner Aspergillus fumigatus via phenazines. Sci. Rep. **5**, 8220 (2015). 25665925 10.1038/srep08220 PMC5389140

[12] J. A. Ferreira , Inhibition of Aspergillus fumigatus and its biofilm by Pseudomonas aeruginosa is dependent on the source, phenotype and growth conditions of the bacterium. PLoS One **10**, e0134692 (2015). 26252384 10.1371/journal.pone.0134692 PMC4529298

[13] E. Mowat , Pseudomonas aeruginosa and their small diffusible extracellular molecules inhibit Aspergillus fumigatus biofilm formation. FEMS Microbiol. Lett. **313**, 96–102 (2010). 20964704 10.1111/j.1574-6968.2010.02130.x

[14] H. Nazik , Novel intermicrobial molecular interaction: Pseudomonas aeruginosa Quinolone Signal (PQS) modulates Aspergillus fumigatus response to iron. Microbiology (Reading) **166**, 44–55 (2020). 31778108 10.1099/mic.0.000858

[15] H. Nazik, G. Sass, E. Deziel, D. A. Stevens, Aspergillus is inhibited by Pseudomonas aeruginosa volatiles. J. Fungi (Basel) **6**, 118 (2020). 32722412 10.3390/jof6030118 PMC7557479

[16] J. C. Penner , Pf4 bacteriophage produced by Pseudomonas aeruginosa inhibits Aspergillus fumigatus metabolism via iron sequestration. Microbiology (Reading) **162**, 1583–1594 (2016). 27473221 10.1099/mic.0.000344

[17] V. V. Phelan , Impact of a transposon insertion in phzF2 on the specialized metabolite production and interkingdom interactions of Pseudomonas aeruginosa. J. Bacteriol. **196**, 1683–1693 (2014). 24532776 10.1128/JB.01258-13 PMC3993319

[18] G. Sass, H. Nazik, P. Chatterjee, D. A. Stevens, Under nonlimiting iron conditions pyocyanin is a major antifungal molecule, and differences between prototypic Pseudomonas aeruginosa strains. Med. Mycol. **59**, 453–464 (2021). 32827431 10.1093/mmy/myaa066

[19] G. Sass , Studies of Pseudomonas aeruginosa mutants indicate pyoverdine as the central factor in inhibition of Aspergillus fumigatus biofilm. J. Bacteriol. **200**, e00345-17 (2018). 10.1128/JB.00345-17 PMC5717155 29038255

[20] F. Shirazi, J. A. Ferreira, D. A. Stevens, K. V. Clemons, D. P. Kontoyiannis, Biofilm filtrates of Pseudomonas aeruginosa strains isolated from cystic fibrosis patients inhibit preformed Aspergillus fumigatus biofilms via apoptosis. PLoS One **11**, e0150155 (2016). 26930399 10.1371/journal.pone.0150155 PMC4773012

[21] K. Keown, A. Reid, J. E. Moore, C. C. Taggart, D. G. Downey, Coinfection with Pseudomonas aeruginosa and Aspergillus fumigatus in cystic fibrosis. Eur. Respir. Rev. **29**, 200011 (2020). 33208485 10.1183/16000617.0011-2020 PMC9489137

[22] R. W. Bastos , Secondary metabolites produced during Aspergillus fumigatus and Pseudomonas aeruginosa biofilm formation. mBio **13**, e0185022 (2022). 35856657 10.1128/mbio.01850-22 PMC9426470

[23] A. Margalit, J. C. Carolan, D. Sheehan, K. Kavanagh, The Aspergillus fumigatus secretome alters the proteome of Pseudomonas aeruginosa to stimulate bacterial growth: Implications for co-infection. Mol. Cell Proteomics **19**, 1346–1359 (2020). 32447284 10.1074/mcp.RA120.002059 PMC8015003

[24] G. Sass , Intermicrobial interaction: Aspergillus fumigatus siderophores protect against competition by Pseudomonas aeruginosa. PLoS One **14**, e0216085 (2019). 31067259 10.1371/journal.pone.0216085 PMC6505954

[25] N. Raffa , Dual-purpose isocyanides produced by Aspergillus fumigatus contribute to cellular copper sufficiency and exhibit antimicrobial activity. Proc. Natl. Acad. Sci. U.S.A. **118**, e2015224118 (2021). 33593906 10.1073/pnas.2015224118 PMC7923669

[26] D. M. Cornforth , Pseudomonas aeruginosa transcriptome during human infection. Proc. Natl. Acad. Sci. U.S.A. **115**, E5125–E5134 (2018). 29760087 10.1073/pnas.1717525115 PMC5984494

[27] D. M. Cornforth, F. L. Diggle, J. A. Melvin, J. M. Bomberger, M. Whiteley, Quantitative framework for model evaluation in microbiology research using pseudomonas aeruginosa and cystic fibrosis infection as a test case. mBio **11**, e03042-19 (2020). 31937646 10.1128/mBio.03042-19 PMC6960289

[28] K. L. Palmer, L. M. Mashburn, P. K. Singh, M. Whiteley, Cystic fibrosis sputum supports growth and cues key aspects of Pseudomonas aeruginosa physiology. J. Bacteriol. **187**, 5267–5277 (2005). 16030221 10.1128/JB.187.15.5267-5277.2005 PMC1196007

[29] K. H. Turner, A. K. Wessel, G. C. Palmer, J. L. Murray, M. Whiteley, Essential genome of Pseudomonas aeruginosa in cystic fibrosis sputum. Proc. Natl. Acad. Sci. U.S.A. **112**, 4110–4115 (2015). 25775563 10.1073/pnas.1419677112 PMC4386324

[30] E. Mowat, J. Butcher, S. Lang, C. Williams, G. Ramage, Development of a simple model for studying the effects of antifungal agents on multicellular communities of Aspergillus fumigatus. J. Med. Microbiol. **56**, 1205–1212 (2007). 17761484 10.1099/jmm.0.47247-0

[31] H. Ostapska , Co-operative biofilm interactions between Aspergillus fumigatus and Pseudomonas aeruginosa through secreted galactosaminogalactan exopolysaccharide. J. Fungi **8**, 336 (2022). 10.3390/jof8040336 PMC9030451 35448567

[32] S. Wurster , Live imaging and quantitative analysis of Aspergillus fumigatus growth and morphology during inter-microbial interaction with Pseudomonas aeruginosa. Virulence **11**, 1329–1336 (2020). 33017225 10.1080/21505594.2020.1827885 PMC7549912

[33] S. J. Wezensky, R. A. Cramer Jr., Implications of hypoxic microenvironments during invasive aspergillosis. Med. Mycol. **49**, S120–S124 (2011). 20560863 10.3109/13693786.2010.495139 PMC2951492

[34] K. Trunk , Anaerobic adaptation in Pseudomonas aeruginosa: Definition of the Anr and Dnr regulons. Environ. Microbiol. **12**, 1719–1733 (2010). 20553552 10.1111/j.1462-2920.2010.02252.x

[35] S. Lhospice , Pseudomonas aeruginosa zinc uptake in chelating environment is primarily mediated by the metallophore pseudopaline. Sci. Rep. **7**, 17132 (2017). 29214991 10.1038/s41598-017-16765-9 PMC5719457

[36] V. G. Pederick , ZnuA and zinc homeostasis in Pseudomonas aeruginosa. Sci. Rep. **5**, 13139 (2015). 26290475 10.1038/srep13139 PMC4542158

[37] R. Furman , DksA2, a zinc-independent structural analog of the transcription factor DksA. FEBS Lett. **587**, 614–619 (2013). 23416301 10.1016/j.febslet.2013.01.073 PMC5525025

[38] M. Gonzalez-Guerrero, D. Raimunda, X. Cheng, J. M. Arguello, Distinct functional roles of homologous Cu+ efflux ATPases in Pseudomonas aeruginosa Mol. Microbiol. **78**, 1246–1258 (2010). 21091508 10.1111/j.1365-2958.2010.07402.x

[39] J. B. Patteson , Biosynthesis of fluopsin C, a copper-containing antibiotic from Pseudomonas aeruginosa. Science **374**, 1005–1009 (2021). 34793213 10.1126/science.abj6749 PMC8939262

[40] D. M. Gardiner, P. Waring, B. J. Howlett, The epipolythiodioxopiperazine (ETP) class of fungal toxins: Distribution, mode of action, functions and biosynthesis. Microbiology (Reading) **151**, 1021–1032 (2005). 15817772 10.1099/mic.0.27847-0

[41] D. H. Scharf , Biosynthesis and function of gliotoxin in Aspergillus fumigatus. Appl. Microbiol. Biotechnol. **93**, 467–472 (2012). 22094977 10.1007/s00253-011-3689-1

[42] H. Seo, S. Kang, Y. S. Park, C. W. Yun, The role of zinc in gliotoxin biosynthesis of Aspergillus fumigatus. Int. J. Mol. Sci. **20**, 6192 (2019). 31817957 10.3390/ijms20246192 PMC6940964

[43] A. M. Traynor , At the metal-metabolite interface in Aspergillus fumigatus: Towards untangling the intersecting roles of zinc and gliotoxin. Microbiology (Reading) **167**, 001106 (2021). 34738889 10.1099/mic.0.001106 PMC8743625

[44] J. W. Bok , LaeA, a regulator of morphogenetic fungal virulence factors. Eukaryot. Cell **4**, 1574–1582 (2005). 16151250 10.1128/EC.4.9.1574-1582.2005 PMC1214197

[45] J. Amich, R. Vicentefranqueira, F. Leal, J. A. Calera, Aspergillus fumigatus survival in alkaline and extreme zinc-limiting environments relies on the induction of a zinc homeostasis system encoded by the zrfC and aspf2 genes. Eukaryot. Cell **9**, 424–437 (2010). 20038606 10.1128/EC.00348-09 PMC2837988

[46] J. W. Bok , GliZ, a transcriptional regulator of gliotoxin biosynthesis, contributes to Aspergillus fumigatus virulence. Infect. Immun. **74**, 6761–6768 (2006). 17030582 10.1128/IAI.00780-06 PMC1698057

[47] A. N. Chan , Role for dithiolopyrrolones in disrupting bacterial metal homeostasis. Proc. Natl. Acad. Sci. U.S.A. **114**, 2717–2722 (2017). 28209778 10.1073/pnas.1612810114 PMC5347557

[48] S. Carberry , Gliotoxin effects on fungal growth: Mechanisms and exploitation. Fungal Genet. Biol. **49**, 302–312 (2012). 22405895 10.1016/j.fgb.2012.02.003

[49] B. Li, C. T. Walsh, Streptomyces clavuligerus HlmI is an intramolecular disulfide-forming dithiol oxidase in holomycin biosynthesis. Biochemistry **50**, 4615–4622 (2011). 21504228 10.1021/bi200321c PMC3101288

[50] J. Jumper , Highly accurate protein structure prediction with AlphaFold. Nature **596**, 583–589 (2021). 34265844 10.1038/s41586-021-03819-2 PMC8371605

[51] C. Wang, S. R. Wesener, H. Zhang, Y. Q. Cheng, An FAD-dependent pyridine nucleotide-disulfide oxidoreductase is involved in disulfide bond formation in FK228 anticancer depsipeptide. Chem. Biol. **16**, 585–593 (2009). 19549597 10.1016/j.chembiol.2009.05.005

[52] M. Schrettl , Self-protection against gliotoxin–A component of the gliotoxin biosynthetic cluster, GliT, completely protects Aspergillus fumigatus against exogenous gliotoxin. PLoS Pathog. **6**, e1000952 (2010). 20548963 10.1371/journal.ppat.1000952 PMC2883607

[53] A. Argyrou, J. S. Blanchard, Flavoprotein disulfide reductases: Advances in chemistry and function. Prog. Nucleic Acid Res. Mol. Biol. **78**, 89–142 (2004). 15210329 10.1016/S0079-6603(04)78003-4

[54] B. Li, W. J. Wever, C. T. Walsh, A. A. Bowers, Dithiolopyrrolones: Biosynthesis, synthesis, and activity of a unique class of disulfide-containing antibiotics. Nat. Prod. Rep. **31**, 905–923 (2014). 24835149 10.1039/c3np70106a PMC4132845

[55] C. Wang, C. J. Flemming, Y. Q. Cheng, Discovery and activity profiling of thailandepsins A through F, potent histone deacetylase inhibitors, from Burkholderia thailandensis E264. Medchemcomm **3**, 976–981 (2012). 23997923 10.1039/C2MD20024D PMC3755959

[56] H. K. Huse , Parallel evolution in Pseudomonas aeruginosa over 39,000 generations in vivo. mBio **1**, e00199-10 (2010). 20856824 10.1128/mBio.00199-10 PMC2939680

[57] S. Freilich , Competitive and cooperative metabolic interactions in bacterial communities. Nat. Commun. **2**, 589 (2011). 22158444 10.1038/ncomms1597

[58] M. E. Hibbing, C. Fuqua, M. R. Parsek, S. B. Peterson, Bacterial competition: Surviving and thriving in the microbial jungle. Nat. Rev. Microbiol. **8**, 15–25 (2010). 19946288 10.1038/nrmicro2259 PMC2879262

[59] D. M. Cornforth, K. R. Foster, Competition sensing: The social side of bacterial stress responses. Nat. Rev. Microbiol. **11**, 285–293 (2013). 23456045 10.1038/nrmicro2977

[60] S. K. Dolan , Regulation of nonribosomal peptide synthesis: Bis-thiomethylation attenuates gliotoxin biosynthesis in Aspergillus fumigatus. Chem. Biol. **21**, 999–1012 (2014). 25126990 10.1016/j.chembiol.2014.07.006

[61] S. D. Zhang, T. Isbrandt, L. L. Lindqvist, T. O. Larsen, L. Gram, Holomycin, an antibiotic secondary metabolite, is required for biofilm formation by the native producer photobacterium galatheae S2753. Appl. Environ. Microbiol. **87**, e00169-21 (2021). 33771780 10.1128/AEM.00169-21 PMC8208159

[62] R. B. Leveson-Gower, C. Mayer, G. Roelfes, The importance of catalytic promiscuity for enzyme design and evolution. Nat. Rev. Chem. **3**, 687–705 (2019).

[63] Y. Liao, G. K. Smyth, W. Shi, The R package Rsubread is easier, faster, cheaper and better for alignment and quantification of RNA sequencing reads. Nucleic Acids Res. **47**, e47 (2019). 30783653 10.1093/nar/gkz114 PMC6486549

[64] M. I. Love, W. Huber, S. Anders, Moderated estimation of fold change and dispersion for RNA-seq data with DESeq2. Genome Biol. **15**, 550 (2014). 25516281 10.1186/s13059-014-0550-8 PMC4302049

[65] L. Glaser , A common approach for absolute quantification of short chain CoA thioesters in prokaryotic and eukaryotic microbes. Microb. Cell Fact **19**, 160 (2020). 32778124 10.1186/s12934-020-01413-1 PMC7418318

[66] A. M. Waterhouse, J. B. Procter, D. M. Martin, M. Clamp, G. J. Barton, Jalview Version 2–A multiple sequence alignment editor and analysis workbench. Bioinformatics **25**, 1189–1191 (2009). 19151095 10.1093/bioinformatics/btp033 PMC2672624

[67] I. Letunic, P. Bork, Interactive Tree Of Life (iTOL) v4: Recent updates and new developments. Nucleic Acids Res. **47**, W256–W259 (2019). 30931475 10.1093/nar/gkz239 PMC6602468

[68] S. Kumar, G. Stecher, M. Li, C. Knyaz, K. Tamura, MEGA X: Molecular evolutionary genetics analysis across computing platforms. Mol. Biol. Evol. **35**, 1547–1549 (2018). 29722887 10.1093/molbev/msy096 PMC5967553

[69] T. Pham, X. Xie, X. Lin, An intergenic “safe haven” region in Aspergillus fumigatus. Med. Mycol. **58**, 1178–1186 (2020). 32171003 10.1093/mmy/myaa009

[70] J. Li, C. Wang, Z. M. Zhang, Y. Q. Cheng, J. Zhou, The structural basis of an NADP(+)-independent dithiol oxidase in FK228 biosynthesis. Sci. Rep. **4**, 4145 (2014). 24553401 10.1038/srep04145 PMC4894451

