# Supplementary material for: Convergent evolution in toxin detection and resistance provides evidence for conserved bacterial–fungal interactions
Source: Proc Natl Acad Sci U S A. 2024 Aug 1;121(32):e2304382121. doi: 10.1073/pnas.2304382121 (PMC11317636; doi:10.1073/pnas.2304382121)
Supplement: Supplementary file 1 — Appendix 01 (PDF) [file pnas.2304382121.sapp.pdf]

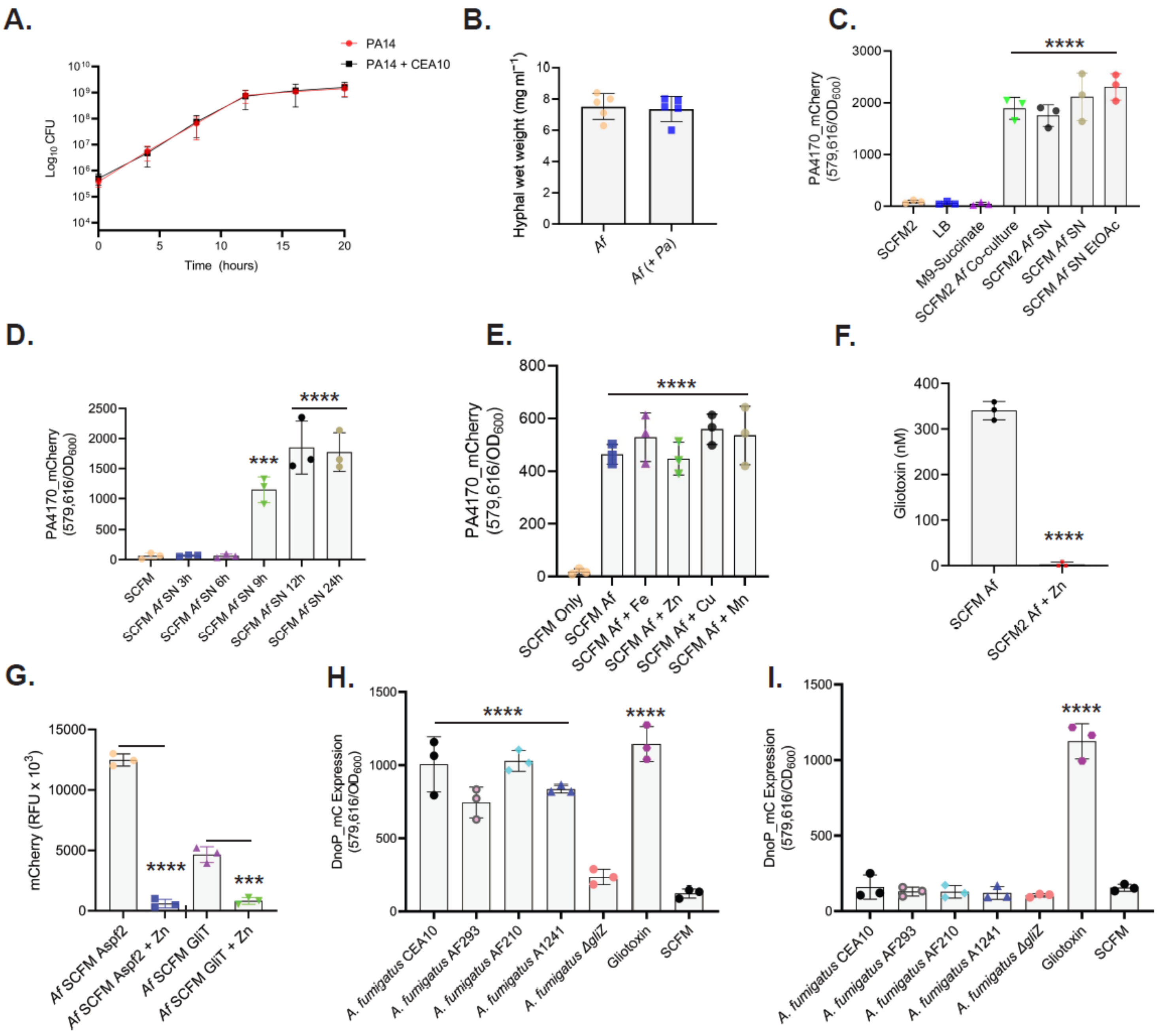

Fig. S1. A. CFUs of *P. aeruginosa* during growth in SCFM2 with and without the presence of *A. fumigatus*. Cultures were plated using the drop-plate method every 4 hr for 20 hr. The data are representative of three independent experiments performed in triplicate. **B.** *A. fumigatus* fungal mass (wet weight mg/ml^{-1}) following 30 hr. growth in SCFM2, with or without *P. aeruginosa* coculture. The data are representative of three independent experiments performed with five replicates. Statistical significance was determined using a Student's t test. **C.** mCherry-tagged PA4170 reporter assay examining PA4170 protein expression following growth in different laboratory media (SCFM, LB, M9-succinate), upon co-culture with *A. fumigatus* (SCFM2 *Pa-Af* co-culture) or using *A. fumigatus* spent culture supernatants (24 hr SCFM/SCFM2). Ethyl-acetate extracted *A. fumigatus* spent

culture supernatants (24 hr) also induce PA4170 expression. Fluorescence was read at 8 hr and normalized to cell density (OD600). The data are representative of three independent experiments performed in triplicate. Statistical significance was calculated by one-way ANOVA with Dunnett's multiple comparisons test using 'SCFM2' as the control column (**** P < 0.0001). Error bars represent standard deviation from the mean. **D.** mCherry-tagged PA4170 reporter fluorescence assay examining PA4170 protein expression following growth in spent *A. fumigatus* culture supernatants grown for 0-24 hr in SCFM. Fluorescence was read at 8 hr and normalized to cell density (OD600). The data are representative of three independent experiments performed in triplicate. Statistical significance was calculated by one-way ANOVA with Dunnett's multiple comparisons test using 'SCFM' as the control column (*** P = 0.0006, **** P < 0.0001). Error bars represent standard deviation from the mean. **E.** mCherry-tagged PA4170 reporter assay examining PA4170 expression following *P. aeruginosa* growth in spent *A. fumigatus* SCFM culture supernatants (24 hr) supplemented with different metals (5 μM $FeSO_4$, $ZnSO_4$, $CuSO_4$, $MnSO_4$) immediately prior to *P. aeruginosa* addition. Fluorescence was read at 8 hr and normalized to cell density (OD600). The data are representative of three independent experiments performed in triplicate. Statistical significance was calculated by one-way ANOVA with Dunnett's multiple comparisons test using 'SCFM only' as the control column (**** P < 0.0001). Error bars represent standard deviation from the mean. **F.** Gliotoxin production by *A. fumigatus* during growth in SCFM2 is repressed by zinc supplementation (10 μM $ZnSO_4$). Gliotoxin was quantified in culture supernatants by LC-MS following 24 hr of *A. fumigatus* growth. The experiment was performed in triplicate. Statistical significance was determined using a Student's t test (**** P < 0.0001). **G.** *A. fumigatus* is zinc limited during growth in SCFM2 and activates expression of the zinc starvation regulated protein Aspf2 and the gliotoxin biosynthetic enzyme GliT. Fluorescence assay examining mCherry-tagged Aspf2 and GliT protein expression during growth in SCFM with or without zinc supplementation (10 μM $ZnSO_4$). Expression was normalized to mCherry expression (+/- 10 μM $ZnSO_4$) under the control of the constitutive *gpdA* promoter. Fluorescence was read at 48 hr. The data are representative of three independent experiments performed in triplicate. Statistical significance was determined using a Student's t test (Aspf2 +/- Zn, GliT +/- Zn) (*** P = 0.0007, **** P < 0.0001). **H.** mCherry-tagged DnoP reporter fluorescence assay examining DnoP protein expression following *P. aeruginosa* growth in spent *A. fumigatus* SCFM culture supernatants (24 h), gliotoxin (30 μM) or SCFM. Fluorescence was read at 8 hr and normalized to cell density (OD600). The data are representative of three independent experiments performed in

triplicate. Statistical significance was calculated by one-way ANOVA with Dunnett's multiple comparisons test using 'SCFM' as the control column (**** $P < 0.0001$). Error bars represent standard deviation from the mean. **I.** mCherry-tagged PA4170 reporter fluorescence assay examining PA4170 protein expression following growth in spent *A. fumigatus* SCFM culture supernatants (24 hr) supplemented with 5 µM $ZnSO_4$ prior to fungal inoculation. Gliotoxin (30 µM) and SCFM wells were also supplemented with 5 µM $ZnSO_4$. Fluorescence was read at 8 hr and normalized to cell density (OD600). The data are representative of three independent experiments performed in triplicate. Statistical significance was calculated by one-way ANOVA with Dunnett's multiple comparisons test using 'SCFM' as the control column (**** $P < 0.0001$). Error bars represent standard deviation from the mean.

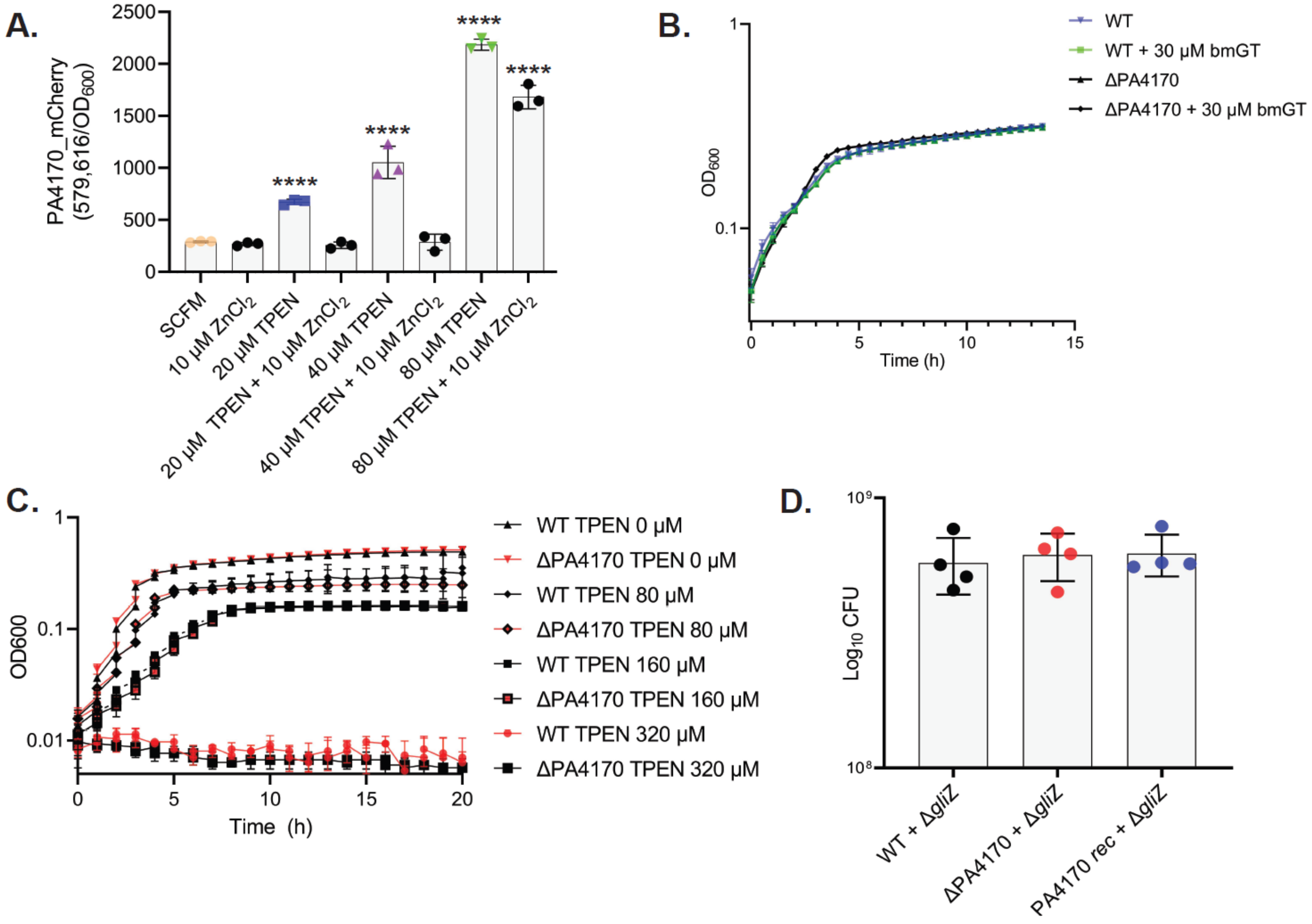

Fig. S2. A. Zinc starvation mediated by TPEN (N,N,N′,N′-tetrakis(2-pyridinylmethyl)-1,2-ethanediamine) supplementation activates *P. aeruginosa* PA4170-mCherry expression in a dose-dependent manner (20-80 µM). Supplementation with 10 µM $ZnSO_4$ partially reverses PA4170 induction. The data are representative of three independent experiments performed in triplicate. Statistical significance was calculated by one-way ANOVA with Dunnett's multiple comparisons test using 'SCFM' as the control column (**** $P < 0.0001$). Error bars represent standard deviation from the mean. **B.** *P. aeruginosa* wild-type or a ΔPA4170 deletion mutant are not susceptible to bisthiomethylgliotoxin exposure (30 µM). Growth curve carried out in MOPS-succinate. The data are representative of three independent experiments performed in triplicate. **C.** Deletion of PA4170 has no impact on the ability of *P. aeruginosa* to grow under TPEN mediated zinc limitation (0-320 µM). Growth curve carried out in SCFM. The data are representative of three independent experiments performed in triplicate. **D.** Growth of a ΔPA4170 deletion mutant is not impacted upon co-culture with *A. fumigatus* Δ*gliZ* when compared to wild type or PA4170 *rec* (plasmid complemented). Cultures were plated for CFU counts 20 hr after *P. aeruginosa* addition. The data are representative of three independent experiments performed in quadruplicate. Statistical significance was calculated by one-way ANOVA with Dunnett's multiple comparisons test using 'WT + Δ*gliZ*' as the control column. Error bars represent standard deviation from the mean.

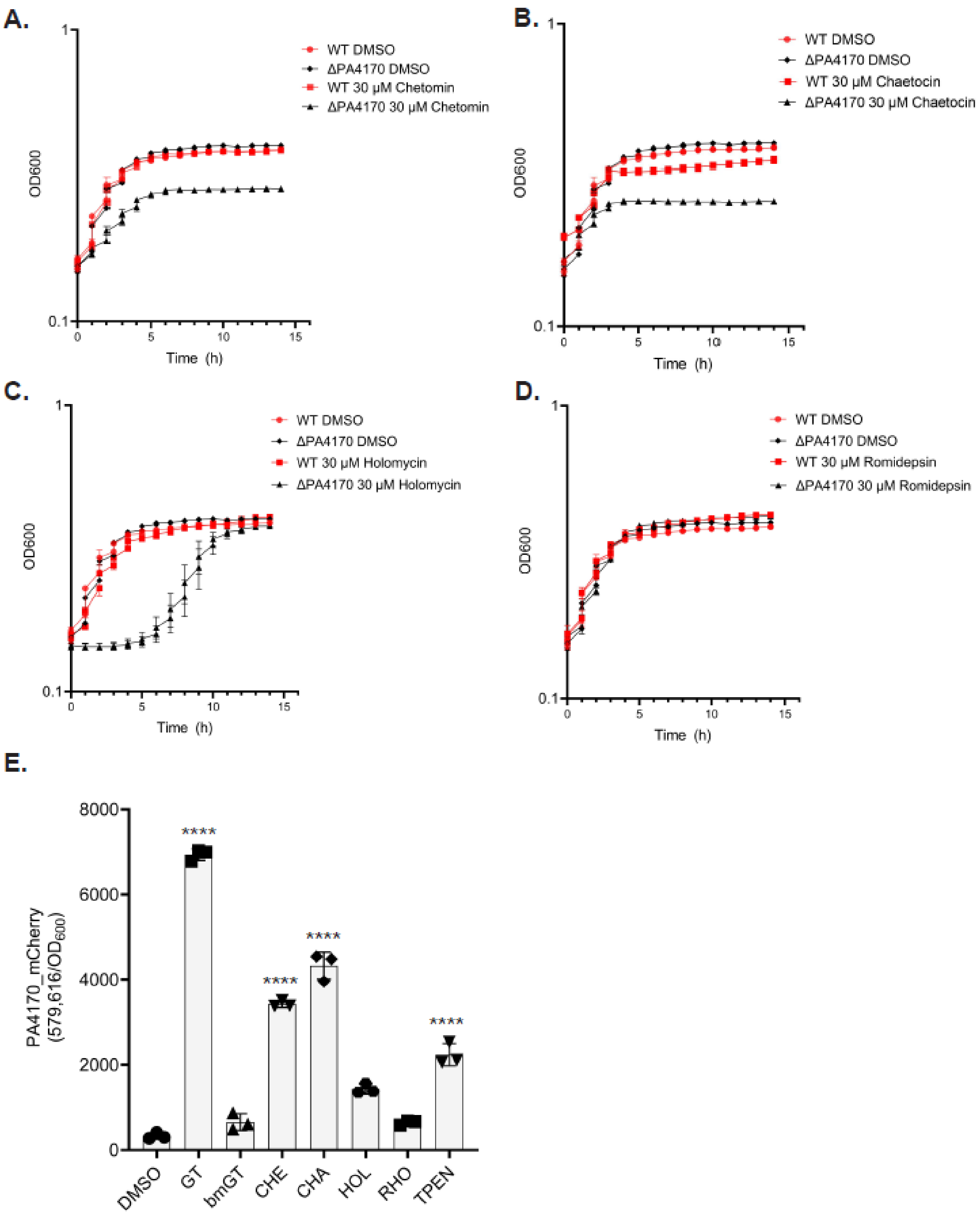

A.
WT DMSO
ΔPA4170 DMSO
WT 30 μM Chetomin
ΔPA4170 30 μM Chetomin
OD600
Time (h)
B.
WT DMSO
ΔPA4170 DMSO
WT 30 μM Chaetocin
ΔPA4170 30 μM Chaetocin
OD600
Time (h)
C.
WT DMSO
ΔPA4170 DMSO
WT 30 μM Holomycin
ΔPA4170 30 μM Holomycin
OD600
Time (h)
D.
WT DMSO
ΔPA4170 DMSO
WT 30 μM Romidepsin
ΔPA4170 30 μM Romidepsin
OD600
Time (h)
E.
PA4170_mCherry (579,616/OD600)
****
DMSO
GT
bmGT
CHE
CHA
HOL
RHO
TPEN

Fig. S3. A. Growth of a ΔPA4170 deletion mutant is more susceptible to chetomin exposure (30 μM) when compared to the *P. aeruginosa* wild type. Strains cultured in M9-succinate. The data are representative of two independent experiments performed in triplicate. **B.** Growth of a ΔPA4170 deletion mutant is more susceptible to chaetocin exposure (30 μM) when compared to the *P. aeruginosa* wild type. Strains cultured in M9-succinate. The data are representative of two independent experiments performed in triplicate. **C.** Growth of a ΔPA4170 deletion mutant is more susceptible to holomycin exposure (30 μM) when compared to the *P. aeruginosa* wild type. Strains cultured in M9-succinate. The data are representative of two independent experiments performed in triplicate. **D.** Romidepsin exposure (30 μM) has no impact on the growth of *P. aeruginosa* wild type or ΔPA4170. Strains cultured in M9-succinate. The data are representative of two independent experiments performed in triplicate. **E**. mCherry-tagged PA4170 reporter assay examining the impact of zinc supplementation (10 μM $ZnSO_4$) on the ability of the ETPs gliotoxin (GT), bisthiomethylgliotoxin (bmGT), chetomin (CHE), chaetocin (CHA), holomycin (HOL) or romidepsin (RHO) to induce PA4170-mCherry expression (all ETPs added at 30 μM). TPEN was added at 50 μM. Fluorescence was read at 8 hr and normalized to cell density (OD600). The data are representative of two independent experiments performed in triplicate. Statistical significance was calculated by one-way ANOVA with Dunnett's multiple comparisons test using 'DMSO' as the control column (**** $P < 0.0001$). Error bars represent standard deviation from the mean.

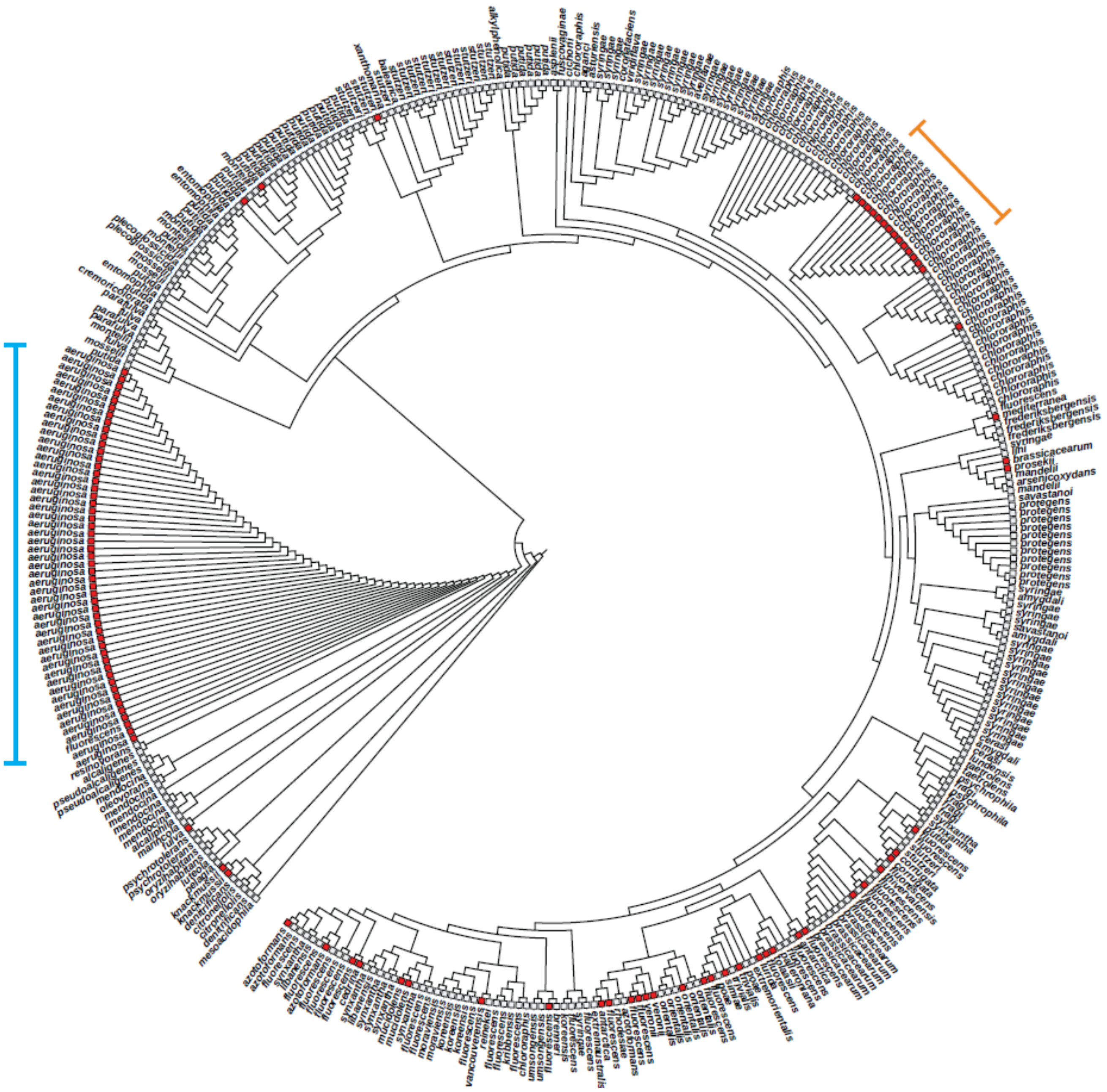

Fig. S4. PA4170 orthologs (red boxes) are found in most sequenced *P. aeruginosa* isolates (279/284) and are interspersed amongst other sequenced *Pseudomonas* strains (n = 74), including *P. fluorescens*, *P. antarctica*, *P. tolaasii* and *P. corrugata*. A previously unidentified phylogenetic sub-branch of *P. chlororaphis* which encodes a PA4170 ortholog is also indicated (orange). Maximum-likelihood tree was assembled with 16s rRNA sequences from representative, sequenced strains across the *Pseudomonas* genus.

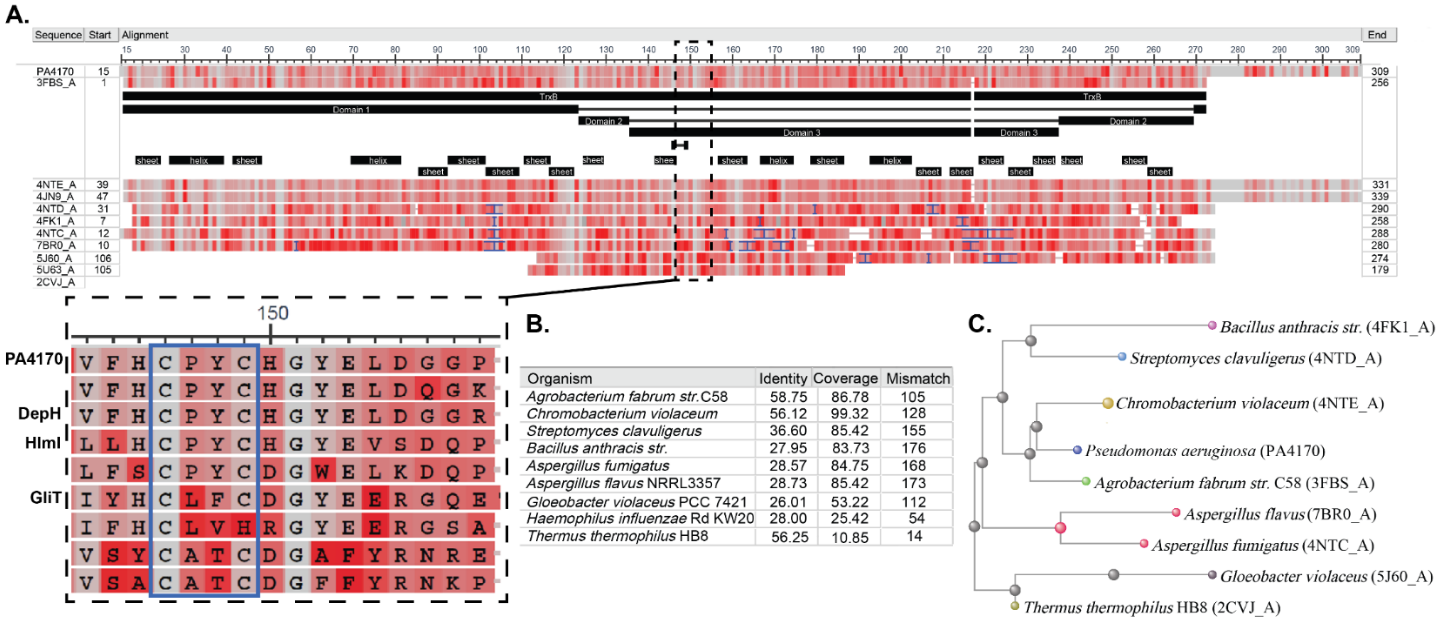

Organism	Identity	Coverage	Mismatch
Agrobacterium fabrum str. C58	58.75	86.78	105
Chromobacterium violaceum	56.12	99.32	128
Streptomyces clavuligerus	36.60	85.42	155
Bacillus anthracis str.	27.95	83.73	176
Aspergillus fumigatus	28.57	84.75	168
Aspergillus flavus NRRL3357	28.73	85.42	173
Gloeobacter violaceus PCC 7421	26.01	53.22	112
Haemophilus influenzae Rd KW20	28.00	25.42	54
Thermus thermophilus HB8	56.25	10.85	14

Fig. S5. A. BLAST search using PA4170 as a query sequence against the Protein Data Bank (PDB) identifies structurally related orthologs of PA4170 in bacteria and fungi– disulfide natural product oxidases (n = 9). Alignments wee visualized with the NCBI Multiple Sequence Alignment Viewer (MSA). The CPYC motif, characteristic of dithiol oxidases is highlighted. **B.** Sequence identity, coverage, and mismatch of the nine BLAST search PDB matches to PA4170. **C.** Phylogenetic tree of PA4170 matches as illustrated by BLAST pairwise alignment tree viewer (Fast Minimum Evolution).

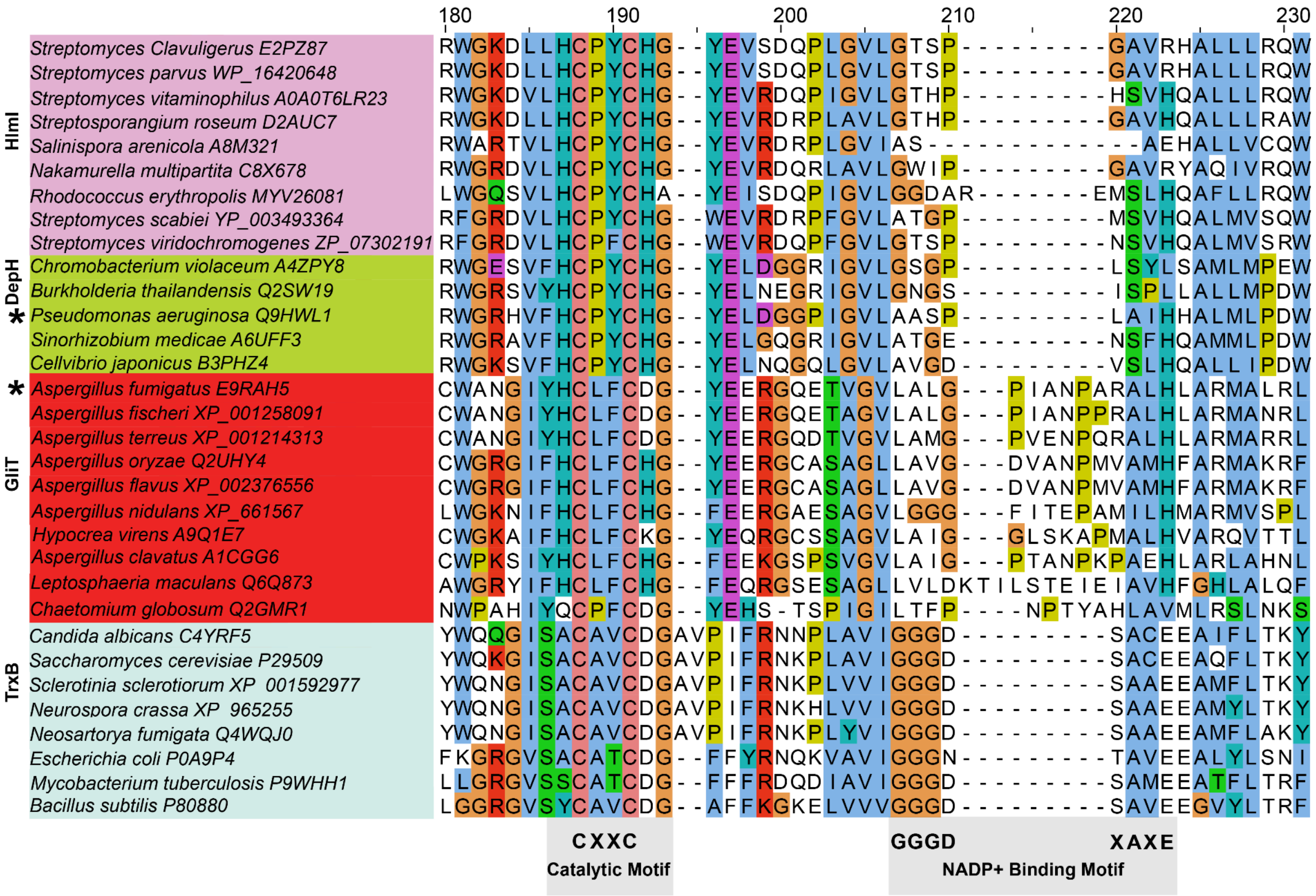

Fig. S6. ClustalW alignment of the catalytic residues in selected bacterial/fungal dithiol oxidases and thioredoxin reductases. The conserved CXXC catalytic motif is indicated, as is the GGGD motif specific for NADP binding in thioredoxin reductases (53). GliT (E9RAH5) and PA4170 (Q9HWL1) are highlighted with asterisks.

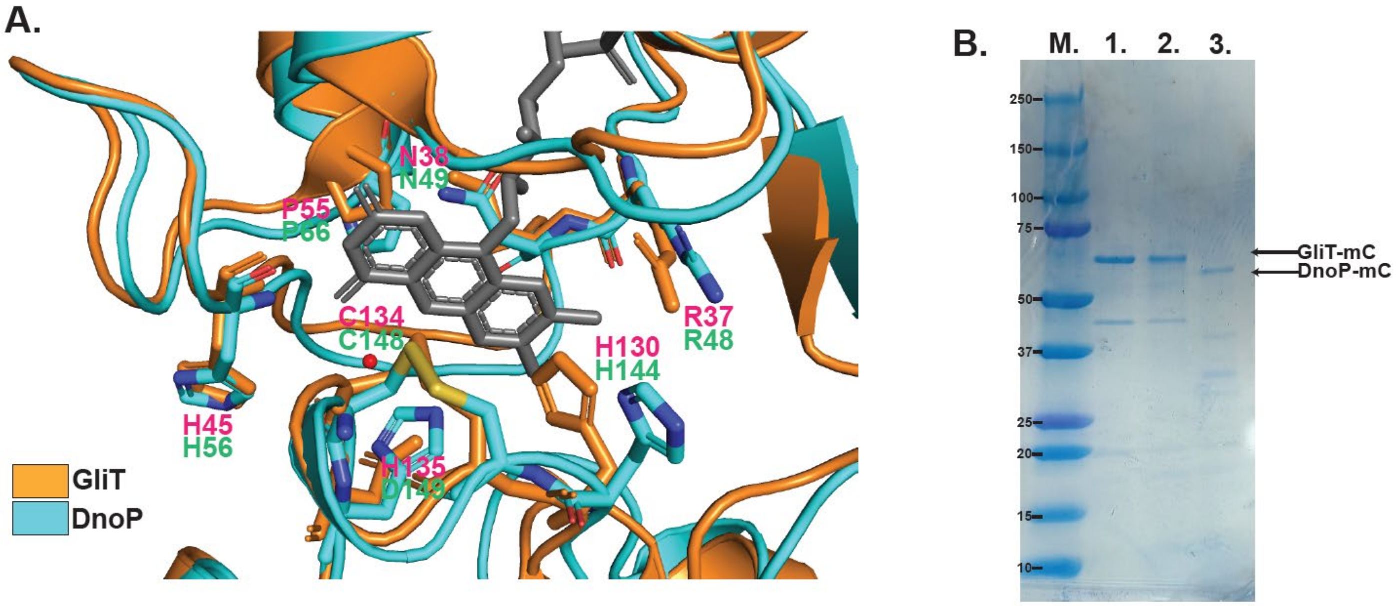

Fig. S7. A. GliT (4NTC) and DnoP (AlphaFold) structure overlay (PyMOL) demonstrating the positions of key shared active site residues (R37/R48, H130/H144, H45/H56, P55/P66, N38/N49, C134/C148) and alterations (H135/D149). FAD shown in grey. **B.** SDS-PAGE gel demonstrating successful ALFA-tag mediated purification of active GliT-mCherry and DnoP-mCherry from *A. fumigatus* strains *gliT* rec and *dnoP* rec, respectively. Lane M; molecular marker. 1; GliT-mCherry A (≈63 kDa), 2; GliT-mCherry B (≈63 kDa), 3; DnoP-mCherry (≈55 kDa).

Table S1. Strains and plasmids used in this study.

Strain name	Description	Source
DH5α	*E. coli* cloning strain	Zymo
BL21(DE3)	*E. coli* protein expression strains	NEB
SM10 (λ pir+)	*E. coli* donor strain for conjugation	(1)
P. aeruginosa PA14	Wild-type laboratory strain	(2)
PA14 Δ*PA4170*	*PA4170* insertion mutant in PA14	This study
PA14 Δ*PA4170 :: PA4170*	PA14 Δ*PA4170* carrying plasmid pUCP20 with PA4170. amp^R	This study
P. aeruginosa PAO1	Wild-type laboratory strain	(3)
P. aeruginosa C5912M	Clinical isolate from CF patient (sputum, mucoid)	(4)
P. aeruginosa C0324C	Clinical isolate from CF patient (sputum, classic)	(4)
PA14 PA4170-mC	*PA4170-ALFA*-mCherry translational fusion in PA14	This study
FGSC A1260 (CEA10)	*A. fumigatus* Wild type: MAT1-1 (also known as CEA10, CBS 144–89CBS 144.89, AF10). Isolated from patient with IPA.	(5)
FGSC A1241	*A. fumigatus* wild type AfIR964	FGSC
FGSC A1259	*A. fumigatus*, clinical isolate AF210 (NCPF 7101)	FGSC
FGSC A1100	*A. fumigatus* wild type AF293	FGSC
A1160 Δ*ku80 pyrG+*	*A. fumigatus* strain derived from A1260 that lacks nonhomologous end joining (*Δku80*). Δ*akuBku80*::*pyrG−-zeo*, *pyrG−*::*pyrGAf*; *MAT1-1,* MFIG001	(6)
A1160 Δ*gliT*::*hygB*	A1160 *gliT* deletion strain, *hygB*	This study
A1160 Δ*gliT*::*hygB; gliT-mC::bleoR*	A1160 *gliT* deletion strain complemented with *gliT-mCherry* (SH1 region), *hygB*:*bleoR*	This study
A1160 Δ*gliT*::*hygB; gliTp-PA4170-mC::bleoR*	A1160 *gliT* deletion strain in A1160 with *gliTp-PA4170-mCherry* inserted (SH1 region), *hygB*:*bleoR*	This study
A1160 Δ*gliZ*::*hygB*	A1160 *gliZ* deletion strain, *hygB*	This study
A1160 *aspf2*-mC*::bleoR*	A1160 with *aspf2-mCherry* inserted in the CEA10 SH1 region, *bleoR*	This study
A1160 *gpdAp*-mC*::bleoR*	A1160 with *gpdAp-mCherry* inserted in the CEA10 SH1 region, *bleoR*	This study

Plasmid name	Description	Source
pUCP20	*E. coli – Pseudomonas* shuttle vector for constitutive expression of cloned genes; Amp^R	(7)
pSKD1	PA4170 promoter and ORF cloned into pUCP20; Amp^R	This study
pET19m	Modified pET19 (Invitrogen), containing TEV protease-cleavable N terminally His6-tag; Amp^R	(8)
pSKD2	*A. fumigatus gliT* ORF cloned into pET-19m; Amp^R	This study
pSKD3	*P. aeruginosa PA4170* ORF cloned into pET-19m; Amp^R	This study
pEXG2	Parental plasmid used markerless DNA mutagenesis; *sacB*, Gen^r	(9)
pSKD4	pEXG2-Δ*PA4170*; *PA4170* deletion cassette; *sacB*, Gen^r	This study
pSKD5	pEXG2-*PA4170-mC; PA4170* mCherry translational fusion cassette; *sacB*, Gen^r	This study
pMF440	Broad host range plasmid for constitutive expression of mCherry; Amp^R	(10)
pAN 7.1	Hygromycin resistant cassette source for PCR amplification; Amp^R	(11)
pAN 8.1	Phleomycin resistant cassette source for PCR amplification; Amp^R	(12)
pSKD6	*gliT* promoter and *gliT* ORF fused with an ALFA tag linker and C-terminus mCherry. Plasmid contains *bleoR* (from pAN 8.1); Amp^R	This study
pSKD7	*gliT* promoter and *PA4170* ORF fused with an ALFA tag linker and C-terminus mCherry. Plasmid contains *bleoR* (from pAN 8.1); Amp^R	This study
pSKD8	*aspf2* promoter and *aspf2* ORF fused with an ALFA tag linker and C-terminus mCherry. Plasmid contains *bleoR (*from pAN 8.1); Amp^R	This study

1. R. Simon, U. Priefer, A. Pühler, A Broad Host Range Mobilization System for In Vivo Genetic Engineering: Transposon Mutagenesis in Gram Negative Bacteria. *Bio/Technology*, 784–791 (1983).
2. L. G. Rahme *et al.*, Common virulence factors for bacterial pathogenicity in plants and animals. *Science* **268**, 1899-1902 (1995).
3. D. M. Cornforth, F. L. Diggle, J. A. Melvin, J. M. Bomberger, M. Whiteley, Quantitative Framework for Model Evaluation in Microbiology Research Using Pseudomonas aeruginosa and Cystic Fibrosis Infection as a Test Case. *mBio* **11** (2020).
4. H. K. Huse *et al.*, Parallel evolution in Pseudomonas aeruginosa over 39,000 generations in vivo. *mBio* **1** (2010).
5. K. McCluskey, The Fungal Genetics Stock Center: from molds to molecules. *Adv Appl Microbiol* **52**, 245-262 (2003).
6. M. Fraczek *et al.*, The cdr1B efflux transporter is associated with non-cyp51a-mediated itraconazole resistance in Aspergillus fumigatus. 1486–1496 (2013).
7. A. Becher, H. P. Schweizer, Integration-proficient Pseudomonas aeruginosa vectors for isolation of single-copy chromosomal lacZ and lux gene fusions. *Biotechniques* **29**, 948-950, 952 (2000).
8. T. Bock, R. Muller, W. Blankenfeldt, Crystal structure of AibC, a reductase involved in alternative de novo isovaleryl coenzyme A biosynthesis in Myxococcus xanthus. *Acta Crystallogr F Struct Biol Commun* **72**, 652-658 (2016).
9. A. Rietsch, I. Vallet-Gely, S. L. Dove, J. J. Mekalanos, ExsE, a secreted regulator of type III secretion genes in Pseudomonas aeruginosa. *Proc Natl Acad Sci U S A* **102**, 8006-8011 (2005).
10. C. Nagant *et al.*, Study of the effect of antimicrobial peptide mimic, CSA-13, on an established biofilm formed by Pseudomonas aeruginosa. *Microbiologyopen* **2**, 318-325 (2013).
11. C. d'Enfert, Selection of multiple disruption events in Aspergillus fumigatus using the orotidine-5'-decarboxylase gene, pyrG, as a unique transformation marker. *Curr Genet* **30**, 76-82 (1996).
12. I. Mattern, P. Punt, C. Van den Hondel, A vector for Aspergillus transformation conferring phleomycin resistance. *Fungal Genet Rep*, 25 (1988).
